# A Dietary Source of High Level of Fluoroquinolone Tolerance in *mcr*-Carrying Gram-Negative Bacteria

**DOI:** 10.34133/research.0245

**Published:** 2023-10-06

**Authors:** Tianqi Xu, Dan Fang, Fulei Li, Zhiqiang Wang, Yuan Liu

**Affiliations:** ^1^ Jiangsu Co-innovation Center for Prevention and Control of Important Animal Infectious Diseases and Zoonoses, College of Veterinary Medicine, Yangzhou University, Yangzhou 225009, China.; ^2^ Joint International Research Laboratory of Agriculture and Agri-Product Safety, the Ministry of Education of China, Yangzhou University, Yangzhou 225009, China.; ^3^ Institute of Comparative Medicine, Yangzhou University, Yangzhou 225009, China.

## Abstract

The emergence of antibiotic tolerance, characterized by the prolonged survival of bacteria following antibiotic exposure, in natural bacterial populations, especially in pathogens carrying antibiotic resistance genes, has been an increasing threat to public health. However, the major causes contributing to the formation of antibiotic tolerance and underlying molecular mechanisms are yet poorly understood. Herein, we show that potassium sorbate (PS), a widely used food additive, triggers a high level of fluoroquinolone tolerance in bacteria carrying mobile colistin resistance gene *mcr*. Mechanistic studies demonstrate that PS treatment results in the accumulation of intracellular fumarate, which activates bacterial two-component system and decreases the expression level of outer membrane protein OmpF, thereby reducing the uptake of ciprofloxacin. In addition, the supplementation of PS inhibits aerobic respiration, reduces reactive oxygen species production and alleviates DNA damage caused by bactericidal antibiotics. Furthermore, we demonstrate that succinate, an intermediate product of the tricarboxylic acid cycle, overcomes PS-mediated ciprofloxacin tolerance. In multiple animal models, ciprofloxacin treatment displays failure outcomes in PS preadministrated animals, including comparable survival and bacterial loads with the vehicle group. Taken together, our works offer novel mechanistic insights into the development of antibiotic tolerance and uncover potential risks associated with PS use.

## Introduction

Antibiotics have transformed modern medicine, and their use has effectively reduced mortality and increased life expectancy. Despite this success, the overuse and misuse of antibiotics in clinics and agricultural production has led to a global antibiotic resistance crisis, severely threatening human and animal health [1]. Colistin is a cationic polypeptide antibiotic that is widely acknowledged as the final option for treating multidrug-resistant bacterial infections, especially caused by carbapenem-resistant Enterobacteriaceae [2]. Nevertheless, the appearance and prevalence of plasmid-borne transmissible colistin resistance gene *mcr-1*, first identified in China, have drastically compromised the clinical efficacy of this last-line antibiotic [3]. Moreover, a range of *mcr-1* variants, encompassing *mcr*-*2* to *mcr*-*10*, have also been identified in different sources of bacterial strains [4]. Nowadays, these mobile colistin resistance genes have been documented in more than 50 countries spanning 6 different continents, posing a marked challenge to public health [5].

Apart from antibiotic resistance, another common but remains understudied bacterial strategy against antibiotic killing is antibiotic tolerance, which allows bacteria to survive longer after being exposed to antibiotics. Antibiotic tolerance is a major contributor to chronic and recurrent infections and is often overlooked in clinical practice owing to the lack of standard quantitative indicators [6]. Furthermore, it has been shown that antibiotic tolerance can promote the evolution of resistance [6–9], even under drug combinations [10], calling for increased attention to tolerance formation. More alarmingly, accumulating studies have revealed the ability of bacteria to develop resistance and tolerance in both clinical and laboratory settings [11,12], leaving no alternatives to clinicians. Therefore, understanding the causes of antibiotic tolerance and underlying molecular mechanisms is of great importance. For example, our previous investigation revealed the relationship between high-fat diet and the development of antibiotic tolerance [13]. In addition, it has been suggested that the loss of some genes, such as *Dsp1* and *Asp23* in *Staphylococcus aureus*, leads to daptomycin tolerance [14], and paradoxical changes in *rpoB* contribute to rifampicin tolerance in mycobacteria [15]. Moreover, previous studies demonstrated that two-component system ZraPSR mediates multidrug tolerance in *Escherichia coli* [16], while antibiotic tolerance in *Mycobacterium tuberculosis* may be due to the CinA protein [17].

**Fig. 1.**
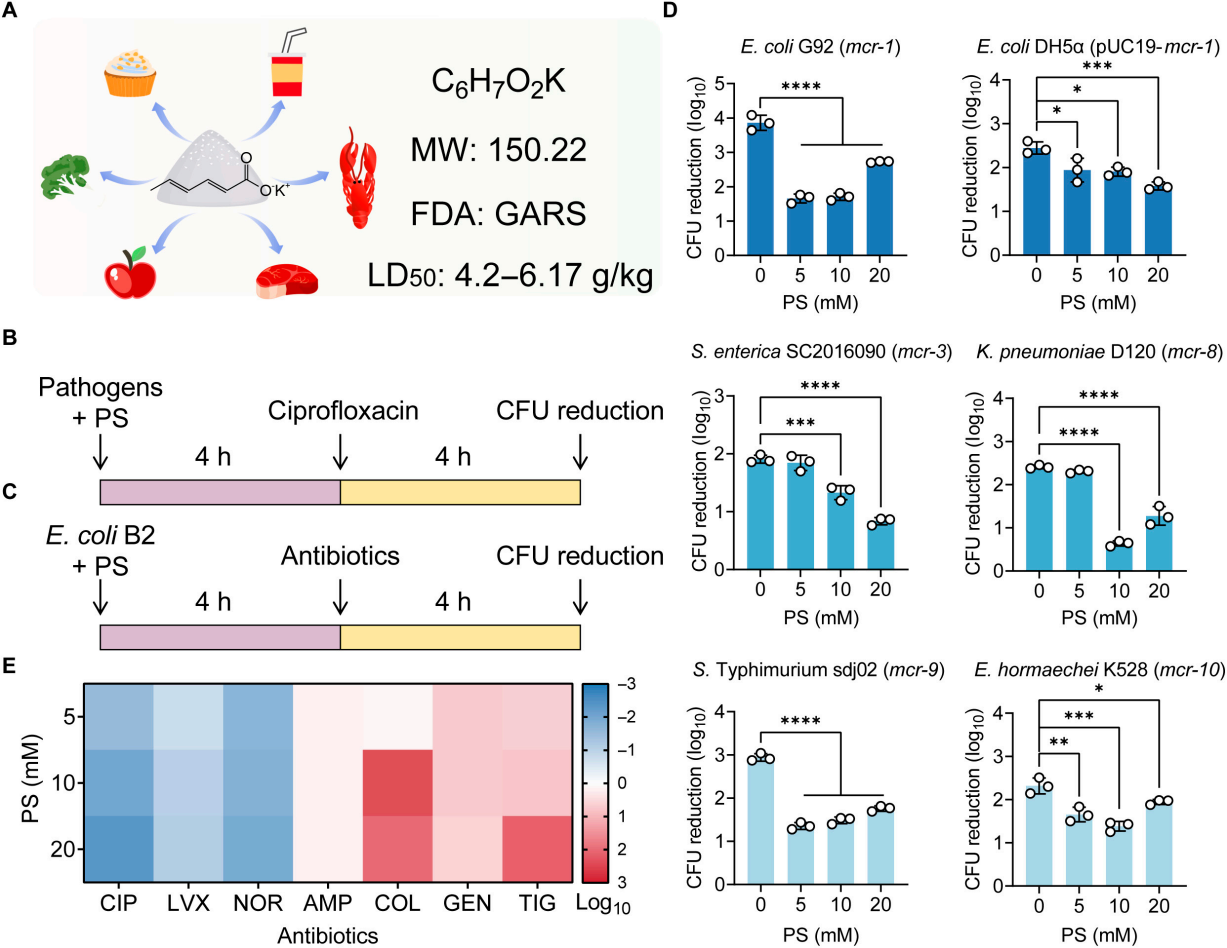
PS reduces the effectiveness of fluoroquinolones against Gram-negative bacteria carrying *mcr*. (A) Skeleton symbol, molecular formula, and molecular weight (MW) of PS. (B) Scheme of the experimental protocols for investigating the effect of PS on ciprofloxacin activity against a panel of *mcr*-positive bacteria. The Gram-negative bacteria carrying *mcr* were cultured with ciprofloxacin (20-fold MIC) for another 4 h after cocultivating with PS for 4 h. (C) Scheme of the experimental protocols for investigating the effect of PS on 7 antibiotics against *E. coli* B2 (*mcr-1*). Cocultivation of PS and *E. coli* B2 for 4 h and then cultured with fluoroquinolones (ciprofloxacin, levofloxacin, and norfloxacin, 40-fold MIC) and other bactericidal antibiotics (ampicillin, colistin, gentamicin, and tigecycline, 40-fold MIC) for another 4 h. (D) PS decreased ciprofloxacin activity against 6 *mcr*-positive bacteria. (E) Relative CFU reduction of *E. coli* B2 (*mcr-1*) after precultured with increasing concentrations of PS (ranging from 0 to 20 mM), which was calculated with the following formula: CFU reduction in the presence of PS − CFU reduction in the absence of PS. The data were shown as means ± SD, and the statistical significance was determined using a nonparametric one-way ANOVA. The levels of significance were denoted as *P < 0.05, **P < 0.01, ***P < 0.001, ****P < 0.0001. FDA, Food and Drug Administration. LD_50_, lethal dose 50.

Potassium sorbate (PS) (Fig. 1A) is a widely used food preservative that could bind to the sulfhydryl groups of microbial enzyme systems, thereby disrupting the action of the enzyme system [18]. PS is generally recognized as safe (GRAS) by the U.S. Food and Drug Administration [19]. The European Food Safety Authority formulates a lower confidence limit of the benchmark dose of 1,110 mg of sorbic acid per kilogram per day and the acute oral lethal dose 50 of PS in rats is 4,200 to 6,170 mg/kg [20]. Previous studies have shown that PS was genotoxic to human peripheral blood lymphocytes [21]. Some studies pointed out that PS had a stimulatory impact on the glycation and fibrillation of human serum albumin and exacerbated the complications of diabetes [22]. A recent study found that PS possessed the ability to modulate the immune system of zebrafish by altering the composition and functionality of the gut microbiota, as well as inhibiting the metabolism of said microbiota [23]. However, despite the extensive use of PS in the food industry, its impact on the efficacy of antibiotic therapy is still elusive.

This study aimed to investigate the potential influence of PS on the formation of antibiotic tolerance in *mcr*-positive superbugs and decipher the underlying molecular mechanisms. Interestingly, we found that PS triggers the development of tolerance to fluoroquinolone antibiotics in *mcr*-positive bacteria both in vitro and in vivo, highlighting a new dietary source of antibiotic tolerance. Mechanistically, PS inhibits aerobic respiration by promoting fumarate accumulation and decreasing antibiotics uptake by regulating bacterial two-component systems. Meanwhile, PS reduces the generation of reactive oxygen species (ROS) and alleviates DNA damage caused by bactericidal antibiotics.

## Results

### PS reduces the bactericidal activity of fluoroquinolone antibiotics against *mcr*-positive bacteria

Prior to evaluating the impact of PS on the effectiveness of antibiotics, we first determined the antibacterial spectrum of PS against different bacterial strains. The results of minimum inhibitory concentration (MIC) analysis showed that PS below 125 mM concentration had no direct inhibitory effect on bacterial growth (Table S2). Next, we characterized the bactericidal activity of ciprofloxacin by calculating colony-forming unit (CFU) reduction after cocultured with PS (Fig. 1B). Interestingly, we found that coincubation of PS with different bacteria carrying *mcr* or its variants remarkably reduced their susceptibility to ciprofloxacin treatment (Fig. 1D). Among them, *Salmonella enterica* SC2016090, *Klebsiella pneumoniae* D120, *Salmonella* Typhimurium sdj02, and *Enterobacter hormaechei* K528 are susceptible to ciprofloxacin (MICs below 0.25 μg/ml) but still showed reduced susceptibility to ciprofloxacin under PS treatment. To exclude the possibility that potassium influenced antibiotic susceptibility, we also tested the effect of sodium sorbate and potassium chloride on the bactericidal activity of ciprofloxacin. The results showed that sorbic acid was responsible for the decrease in bactericidal activity, while potassium alone had no effect (Fig. S1). Given that ciprofloxacin is a bactericidal antibiotic that binds to DNA gyrase and DNA topoisomerase to disrupt DNA replication and transcription, we set out to test whether PS could also reduce the effect of other bactericidal antibiotics. We selected 4 antibiotics, including β-lactam antibiotic ampicillin, which binds penicillin-binding proteins to inhibit cell wall biosynthesis; cationic antimicrobial peptide colistin, which binds primarily to lipopolysaccharides in bacterial outer membrane; aminoglycosides antibiotic gentamicin, which binds to the 30S ribosomal subunit and induces protein mistranslation, and tetracycline antibiotic tigecycline, which binds bacterial 30S ribosomal subunit components (16S ribosomal RNA) to block protein translation (Fig. 1C). However, we found that PS did not affect the sterilization effect of these antibiotics (Fig. 1E). Considering these findings, we hypothesized that PS might specifically hinder the effectiveness of fluoroquinolone antibiotics against *mcr*-positive bacteria. To verify our hypothesis, we next tried 2 other typical fluoroquinolone antibiotics, levofloxacin and norfloxacin. Consistently, PS also reduced their bactericidal effect against *E. coli* B2 (Fig. 1E). Taken together, these data suggest that PS attenuates the activity of fluoroquinolone antibiotics against Gram-negative bacteria carrying *mcr* gene.

### PS triggers high level of antibiotic tolerance instead of antibiotic resistance or persistence

**Fig. 2.**
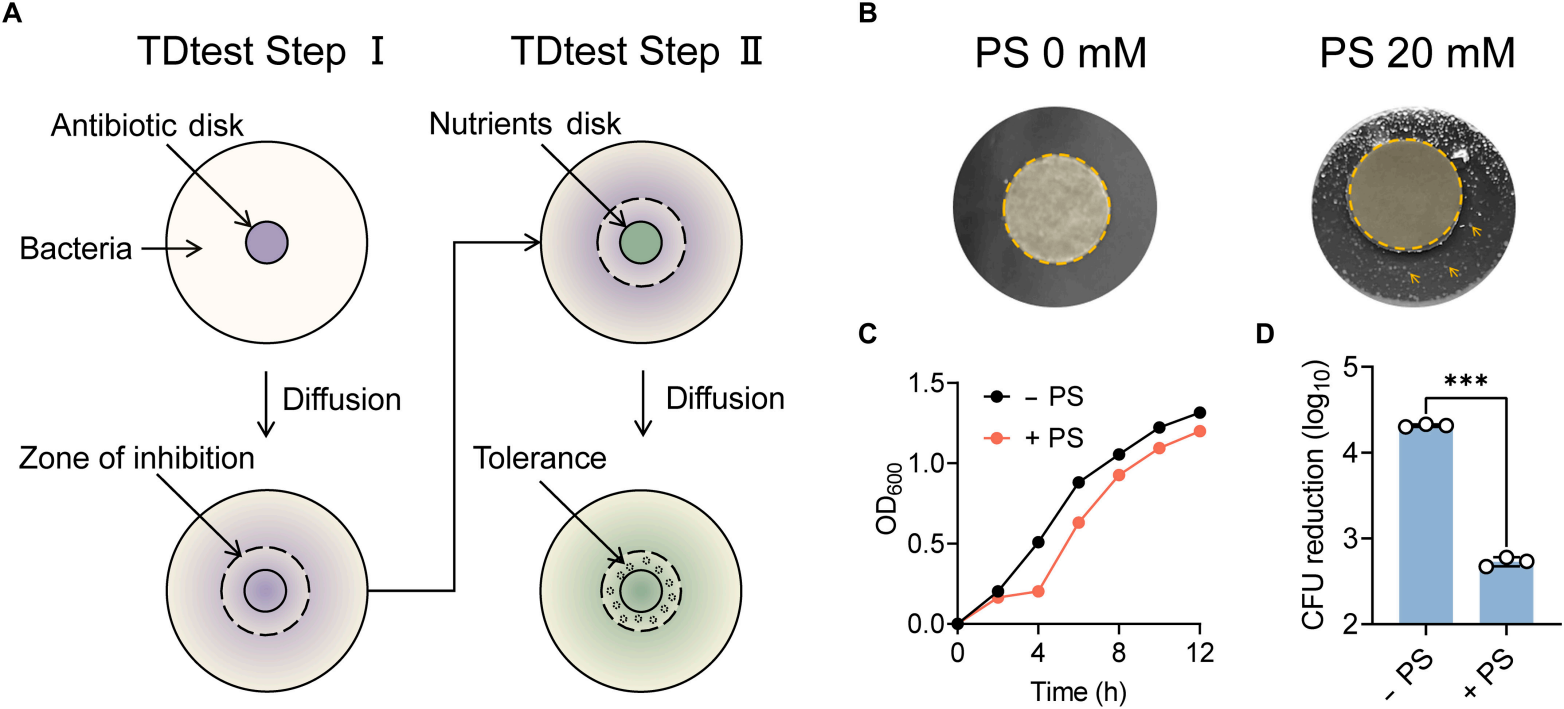
Isolation and identification of tolerance strains induced by PS using the TDtest. (A) Scheme of the experimental protocols for TDtest. TDtest Step I: Place the antibiotic disk on the agar plate before spreading *E. coli* B2 on the agar. The antibiotic spread across the whole plate and the zone of inhibition appear within 18 h. TDtest Step II: Replace antibiotic disk with nutrition disk after antibiotic concentration decreases below MIC. The nutrition diffuses through the plate, and the tolerant strains grow inside the inhibition zone. (B) Tolerance detection using the TDtest. Left: *E. coli* B2 was not cocultured with PS before spreading on plate. After glucose addition, there is no late growth. Right: Before spreading on plates, the bacteria were treated with PS (20 mM) for 4 h*.* The tolerant bacteria are those within the inhibition zone after glucose addition. The arrows pointed to the late-appearing bacteria grown in the inhibition zone. (C) The growth curve of a single colony selected randomly from TDtest. (D) CFU reduction (log_10_) of initial *E. coli* B2 and tolerant strain after treatment with ciprofloxacin. The data were shown as means ± SD, and the statistical significance was determined using a nonparametric 1-way ANOVA. The levels of significance were denoted as ***P < 0.001.

Considering that the decreased susceptibility of bacteria to antibiotics may be due to the development of resistance and whether bacteria are resistant to antibiotics can be judged by the MIC, we tested the MICs of different bacteria to ciprofloxacin after coincubating with PS for 4 h (Table S4). The findings showed that the MIC values for ciprofloxacin remained unchanged both before and after PS induction, demonstrating that PS did not induce heritable antibiotic resistance. Next, we used tolerance detection tests (TDtests) to explore whether PS can lead to antibiotic tolerance (Fig. 2A) [24]. Interestingly, there were colonies grew inside the zone of inhibition after glucose addition (Fig. 2B), indicating slow- or late-growing bacteria of *E. coli* B2 (*mcr-1*) after cocultured with PS. Then, single colonies were selected randomly for further analysis. MIC analysis (Table S5), growth curves (Fig. 2C), and killing assays (Fig. 2D) conducted on these clones demonstrated that the late-appearing phenotype exhibited diminished susceptibility to antibiotic-induced killing and slow growth but did not exhibit any alteration in MIC values, illustrating that PS induced tolerance or persistence. To further distinguish it, we determined the effect of PS on MDK_99_, namely, the minimum length of time for killing 99% of the bacterial population. Results showed that PS incubation delayed the timing of MDK_99_ by 2.5 h (Fig. S2). Considering that the MDK_99_ of the persistent bacteria did not change compared with the susceptible bacteria, we concluded that PS triggered antibiotic tolerance. Further whole-genome sequencing analysis identified a few single-nucleotide polymorphisms related to phage protein, transposase, and translation elongation factor (Table S7). Tolerance strains showed no change in resistance to sulfamethoxazole and chloramphenicol (Table S5), despite the presence of sulfonamide resistance protein and chloramphenicol/florfenicol resistance-associated single-nucleotide polymorphisms. These results indicate that PS-induced reduced bacterial susceptibility to fluoroquinolones is only a phenotypic change, that is, antibiotic tolerance rather than drug resistance or persistence.

### Transcriptome analysis of *mcr*-carrying *E. coli* after exposure to PS

**Fig. 3.**
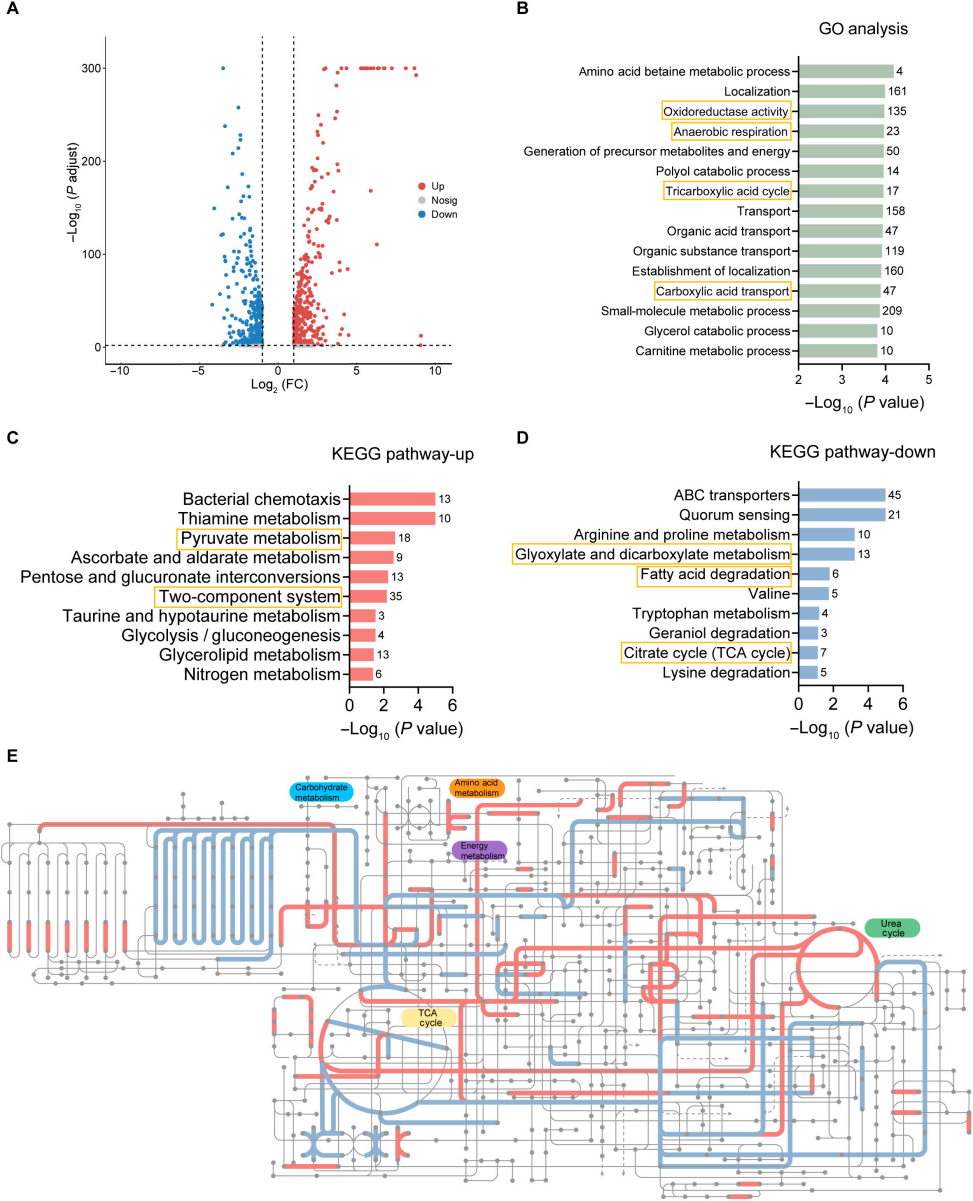
Transcriptome analysis of *E. coli* carrying *mcr* upon treatment with PS. (A) Volcano plot of differently expressed genes. Log_2_ fold changes (Log_2_(FC)) in mRNA in *E. coli* G92 versus *E. coli* G92 treated with PS and the corresponding significance values were displayed as log_10_ (*P* value). (B to D) Functional enrichment analysis of genes, including GO enrichment (B) and KEGG enrichment (C and D). (E) Ipath analysis for global metabolic flux. Analyses of the DEGs resulting from *E. coli* G92 compared with *E. coli* G92 treated with PS. IPath3.0 is used to further analyze metabolic network pathways in *E. coli* G92. Blue and red lines represent the decrease and increase gene annotation pathway, respectively.

To dissect the underlying mechanisms by which PS triggers a high level of fluoroquinolone tolerance, we performed transcriptome analysis of *E. coli* G92 (*mcr-1*) in the presence or absence of PS (10 mM) for 4 h. Principal component analysis revealed significant differences between PS-treated and untreated groups. Specifically, PS treatment induced a series of differentially expressed genes (DEGs), among which 431 genes were up-regulated and 358 genes were down-regulated (fold change ≥ 2-fold, Fig. 3A). Gene Ontology (GO) enrichment analysis revealed 404 GO biological processes, mainly involved in oxidoreductase activity, anaerobic respiration, tricarboxylic acid (TCA) cycle, and carboxylic acid transport (Fig. 3B). Consistently, Kyoto Encyclopedia of Genes and Genomes (KEGG) enrichment analysis showed that these up-regulated DEGs were mainly related to pyruvate metabolism and two-component system (Fig. 3C), while these down-regulated DEGs were involved in glyoxylate and dicarboxylate metabolism, fatty acid degradation, and TCA cycle (Fig. 3D). To investigate the impact of PS on bacterial metabolism more intuitively, we used iPath3.0 (https://pathways.embl.de/) to visualize the key metabolic pathways (Fig. 3E). iPath analysis showed that the PS treatment altered numerous metabolic pathways, of which carbohydrate metabolism and energy metabolism were the most important. In summary, our results suggest that PS-mediated antibiotic tolerance is linked to metabolic changes in *mcr*-positive bacteria.

### PS weakens bacterial metabolism by inhibiting aerobic respiration

**Fig. 4.**
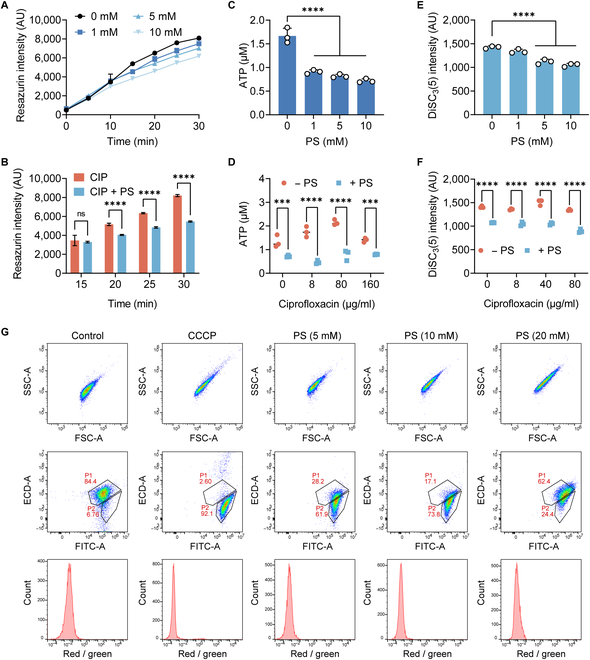
PS restrains bacterial metabolism. (A and B) Bacterial respiration levels of *E. coli G92* after incubated with PS (ranging from 0 to 10 mM, [A]), ciprofloxacin (8 μg/ml), or in combination with PS (10 mM, [B]) within 30 min, determined using resazurin. (C and D) Intracellular ATP concentrations in *E. coli G92* following exposure to varying concentrations of PS (ranging from 0 to 10 mM, [C]), ciprofloxacin (ranging from 0 to 160 μg/ml), or in conjunction with PS (10 mM, [D]). (E and F) DiSC_3_(5) fluorescence changes in *E. coli* G92 upon exposure to PS (ranging from 0 to 10 mM, [E]), ciprofloxacin (ranging from 0 to 160 μg/ml), or combined with PS (10 mM, [F]). (G) Bacterial PMF of *E. coli* G92 after cocultured with PS. Carbonyl cyanide 3-chlorophenylhydrazone (CCCP) was used as a PMF dissipator. The data were shown as means ± SD, and the statistical significance was determined using a nonparametric one-way ANOVA. The levels of significance were denoted as **P < 0.01, ***P < 0.001, ****P < 0.0001. ns, not significant; FITC, fluorescein isothiocyanate; ECD, electron coupled dye; AU, arbitrary unit; SSC, side scatter; FSC, forward scatter.

The bactericidal effect of antibiotics is strongly influenced by the metabolic condition of bacteria [25]. Respiration can oxidize and decompose organic matter to release energy for metabolism, especially aerobic respiration. From the results of transcriptomics, we found that the transcription levels of genes related to aerobic respiration (*sdhA*, *sdhB*, *nuoA*, *cyoB*, *cyoC*, and *cyoD*) were significantly reduced (Fig. 3A). Simultaneously, genes related to the anaerobic respiratory chain with fumarate as the terminal electron acceptor were significantly up-regulated (Fig. 3A). The DcuA and DcuB carrier proteins are responsible for the exchange of 4-carbon (C4) dicarboxylic acids (including succinate, fumarate, malate, and aspartate) in and out of the membrane under anaerobic conditions [26]. After C4-dicarboxylates are transported into the cell membrane, the TCA cycle proceeds reversibly [27], in which *fumB* promotes the conversion of malate to fumarate [28], and *frdABCD* (*FRD*) converts fumarate to succinate [29]. These results suggested that the inclusion of PS might have an impact on bacterial respiration. In order to evaluate this, we proceeded to analyze the impact of PS on bacterial aerobic respiration using a redox indicator resazurin. The results showed that PS significantly inhibited bacterial aerobic respiration, with the extent of inhibition being dependent on the dosage (Fig. 4A), and could antagonize the stimulatory impact of ciprofloxacin on bacterial respiration (Fig. 4B). We also used the R01 fluorescent probe to evaluate the oxygen consumption of *E. coli* G92. Compared with the untreated group, the oxygen consumption of PS-treated bacteria was significantly reduced (Fig. S3), similar to the bacteria in the anaerobic respiration. Adenosine triphosphate (ATP), the product of the electronic respiratory chain, is the direct source of energy required for bacterial metabolism. Thus, we detected the levels of ATP within the cells following PS exposure. We found that PS alone decreased the amount of ATP (Fig. 4C) and prevented ATP increase by ciprofloxacin (Fig. 4D). Considering that bacterial proton motive force (PMF) is necessary for the synthesis of ATP through the F1F0-adenosine triphosphatase, we hypothesized that PS may dissipate PMF, which comprises membrane potential (ΔΨ) and transmembrane proton gradient (ΔpH). To test this hypothesis, we applied a fluorescence probe 3,3′-dipropylthiadicarbocyanine iodide (DiSC_3_(5)), which can accumulate in the cytoplasmic membrane and be released into the extracellular environment leading to increased fluorescence when ΔΨ is disrupted. In contrast, the dissipation of ΔpH would enhance the uptake of DiSC_3_(5) and result in decreased fluorescence. Consequently, we observed a dose-dependent decrease of fluorescence units (Fig. 4E), regardless of the presence of ciprofloxacin (Fig. 4F), indicating that PS specifically dissipated the ΔpH component of bacterial PMF. Furthermore, we measured the overall effect of PS on bacterial PMF by flow cytometry using 3,3′-diethyloxa-carbocyanine iodide (DiOC_2_(3)) fluorescent dye. Notably, we found that PS, especially at 10 mM, drastically disrupted bacterial PMF (Fig. 4G), potentially leading to a reduction in ATP synthesis. These findings indicate that the addition of PS weakens bacterial metabolism by inhibiting bacterial aerobic respiration and reduces ATP production by dissipating bacterial PMF, thereby impairing ciprofloxacin activity.

### PS leads to accumulation of fumarate and activation of two-component systems

**Fig. 5.**
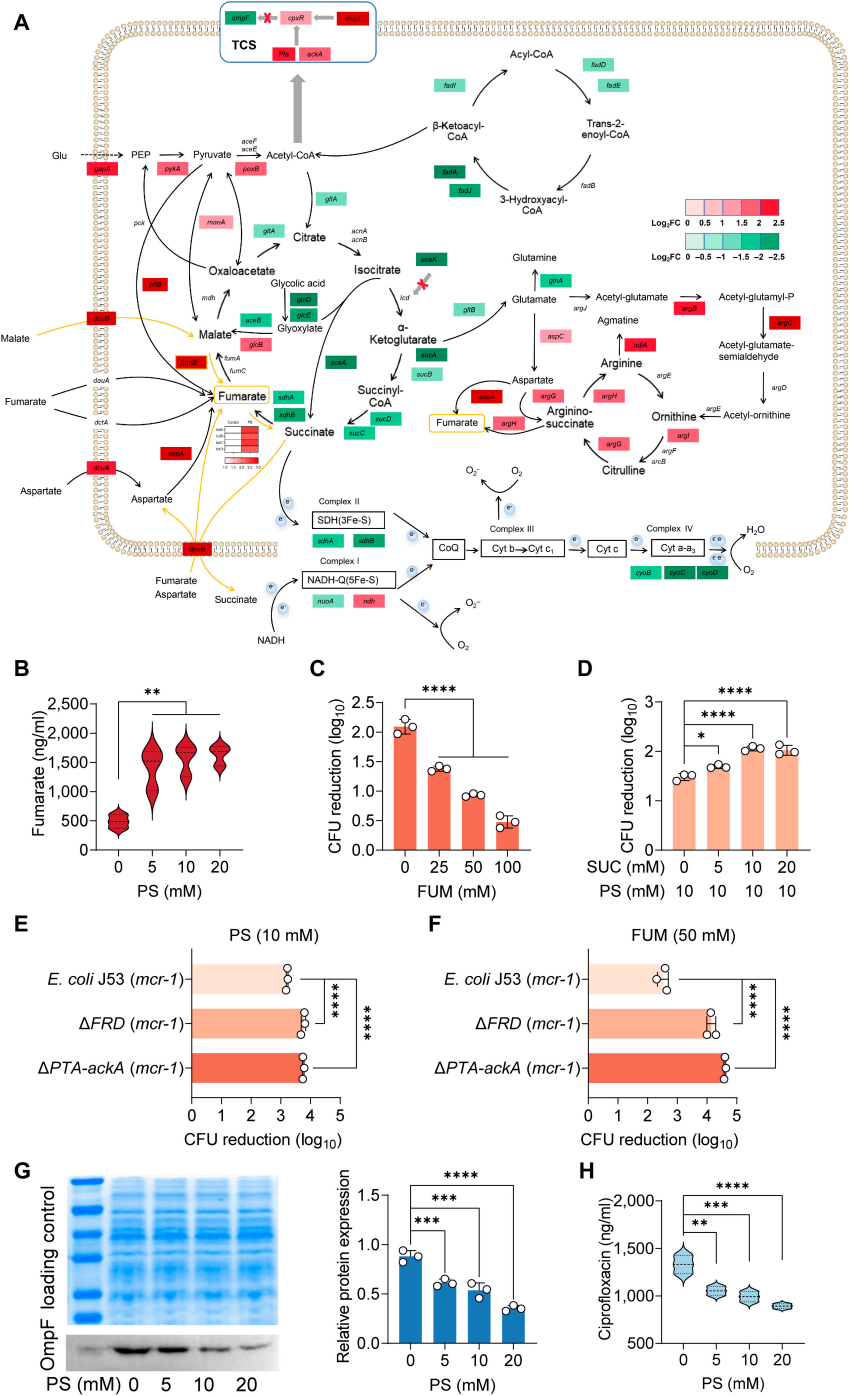
PS promotes intracellular fumarate accumulation through the paired *FRD/SDH* System. (A) Selected DEGs involved in the pyruvate cycle, fatty acid degradation electron transport chain, amino acid metabolism, and two-component regulatory system. Color shades indicate log_2_ fold changes of increased (red) or decreased (green) gene expression under exposure of PS. (B) PS dose-dependently increases the accumulation of intracellular fumarate in a dose-dependent manner. (C) Fumarate supplementation decreases the bactericidal activity of ciprofloxacin against *E. coli* G92. *E. coli* G92 was cultivated in the presence of fumarate (ranging from 0 to 100 mM) for 4 h and treated with ciprofloxacin (20-fold MIC) for another 4 h; the reduction of CFUs was calculated. (D) The addition of succinate (SUC) abolishes the ciprofloxacin tolerance induced by PS and improves ciprofloxacin activity against *E. coli* G92. *E. coli* G92 were cultivated in the presence of succinate (ranging from 0 to 20 mM) and PS (10 mM) for 4 h and treated with ciprofloxacin (20-fold MIC) for another 4 h. (E and F) Deletion of *FRD* or *PTA-ackA* strongly attenuates the effect of PS (E) or fumarate (F) exposure on ciprofloxacin activity. (G) Western blot analysis for OmpF protein of *E. coli* G92 in the presence of increasing concentrations of PS (ranging from 0 to 20 mM) for 4 h. The relative protein expression normalized to the loading control was calculated accordingly. (H) PS decreases the accumulation of intracellular ciprofloxacin in a dose-dependent manner. The data were shown as means ± SD, and the statistical significance was determined using a nonparametric one-way ANOVA. The levels of significance were denoted as *P < 0.05, **P < 0.01, ***P < 0.001, ****P < 0.0001.

To explore the molecular mechanisms underlying PS-induced tolerance, combined with transcriptomics, we focused on pathways related to carbon metabolism and energy metabolism (Fig. 5A). After PS treatment, genes related to lipid metabolism and pyruvate cycle were significantly down-regulated. However, fumarate-related genes, such as *fumB* mediating the conversion of malate into fumarate and *FRD* gene mediating the reduction of fumarate to succinate, were significantly up-regulated. Meanwhile, the transcript levels of *aspA* and *argH*, accounting for the production of fumarate from aspartate and arginino-succinate, were also markedly enhanced. These changes implied that the addition of PS may result in a rise in the content of fumarate. Thus, we measured the intracellular fumarate accumulation by liquid chromatography-tandem mass spectrometry (LC-MS/MS) analysis and observed a significant elevation of intracellular fumarate content in response to PS in a dose-dependent manner (Fig. 5B). Fumarate is one of the C4-dicarboxylic acids, which is produced by the oxidation of succinate as an intermediate in the TCA cycle. A previous study reported that the persistence frequency of *E. coli* is associated with intracellular fumarate [30]. Considering these discoveries, we hypothesized that the accumulated fumarate might have a crucial impact on the PS-induced high level of antibiotic tolerance. To test this, we investigated whether fumarate supplementation could also induce antibiotic tolerance. After fumarate was added, CFU reduction decreased in a dose-dependent manner (Fig. 5C). Moreover, the addition of fumarate led to the weakening of bacterial metabolism, which was manifested by a dose-dependent decrease in ATP (Fig. S4B) and respiration level (Fig. S4C). Consistently, the addition of fumarate also decreased bacterial PMF (Fig. S4E and F). These phenomena were in agreement with the supplementation of PS; thus, we concluded that PS can induce fumarate accumulation, leading to the inhibition of aerobic respiration and decreased ATP level.

Considering that the expression of *FRD* and *sdhCDAB* (*SDH*) genes is usually coupled, up-regulation of *SDH* would reduce the transcription of *FRD* [30], thereby inhibiting the anaerobic respiratory chain with fumarate as the terminal electron acceptor and reducing the utilization of fumarate. It is suggested that both FRD and succinate dehydrogenase (SDH) possess the capability to reduce fumarate and oxidized succinate, and the addition of succinate would activate SDH [30]. Consistently, our results showed that PS or fumarate supplementation clearly reduced SDH activity (Fig. S5A and B), whereas this phenomenon was prevented by adding succinate (Fig. S5C). Furthermore, the addition of succinate abolished the inhibitory effect of PS on antibiotic activity (Fig. 5D). To further verify the role of fumarate, we constructed the deletion mutants of fumarate reductase (Δ*FRD*) in engineered *E. coli* J53 carrying *mcr-1* plasmid. The results showed that PS- or fumarate-mediated antibiotic tolerance was completely reversed in Δ*FRD* strain due to impaired fumarate utilization (Fig. 5E and F). Preincubation with PS or fumarate could even enhance the bactericidal activity of ciprofloxacin (Fig. S6), which supports our idea that PS promoted the accumulation and utilization of fumarate, thereby resulting in the increased expression of fumarate reductase.

Unlike other terminal electron acceptors, fumarate is also an important carbon source. When carbon sources are in excess, the Pta-AckA pathway is activated, which generates acetyl phosphate groups from acetyl-coenzyme A (CoA) to phosphorylate CpxR [31]. Consistently, our results demonstrated that the bactericidal effect of ciprofloxacin on Δ*Pta-AckA* strain was enhanced compared with original *E. coli* J53 (*mcr-1*) despite the presence of PS or fumarate (Fig. 5E and F and Fig. S6), indicating that Pta-AckA pathway is indeed associated with PS-meditated tolerance. Also, similar results were observed in a *FRD* and *PTA-ackA* double mutant, which did not achieve ciprofloxacin tolerance after coincubation with PS or fumarate (Fig. S6). CpxR belongs to the Cpx two-component system, which has certain cross-regulation with other two-component regulatory systems; for example, EnvZ/OmpR can be activated by activating Cpx [32]. The binary regulatory systems EnvZ/OmpR and CpxA/CpxR finely regulate the expression level of outer membrane protein F (OmpF). Specifically, the activation (phosphorylation) of CpxR would decrease the expression of OmpF [33]. Thus, we speculated that the accumulation of fumarate may result in a reduction in the OmpF gene expression. Next, we conducted quantitative reverse transcription-polymerase chain reaction (RT-qPCR) and Western blot analysis to test this hypothesis. As expected, we found that the transcription (Fig. S7) and translation levels of OmpF protein were significantly reduced by PS or fumarate in a dose-dependent manner (Fig. 5G and Fig. S4D). Previous studies have shown that the entry of fluoroquinolones into bacterial cells is mainly through OmpF, and the absence of OmpF is one of the reasons for the decreased susceptibility of *E. coli* to fluoroquinolones [34–37]. To verify it, we constructed an OmpF knockout strain in *E. coli* J53 (*mcr-1*) and assessed the antibacterial activity of ciprofloxacin. Compared with the nonknockout strain, the MIC of ciprofloxacin in Δ*OmpF* strain was not changed (Table S3). However, we found that Δ*OmpF* strain exhibited noticeable tolerance to ciprofloxacin without PS or fumarate pretreatment (Fig. S8), which further validated our hypothesis that OmpF expression is of great importance for ciprofloxacin activity. Moreover, EtBr analysis showed that PS mildly enhanced bacterial efflux pump function (Fig. S9). Based on these points, we measured the intracellular ciprofloxacin content of *E. coli* G92 subjected to varying concentrations of PS by LC-MS/MS analysis. As a consequence, our findings indicated that with the increase of PS concentration, the intracellular accumulation of ciprofloxacin was significantly reduced (Fig. 5H). These results indicate that PS activates bacterial two-component system through accumulating fumarate and then reduces the expression of membrane protein OmpF, thereby reducing the intracellular accumulation of drugs.

### PS reduces ciprofloxacin-induced ROS generation and DNA damage

**Fig. 6.**
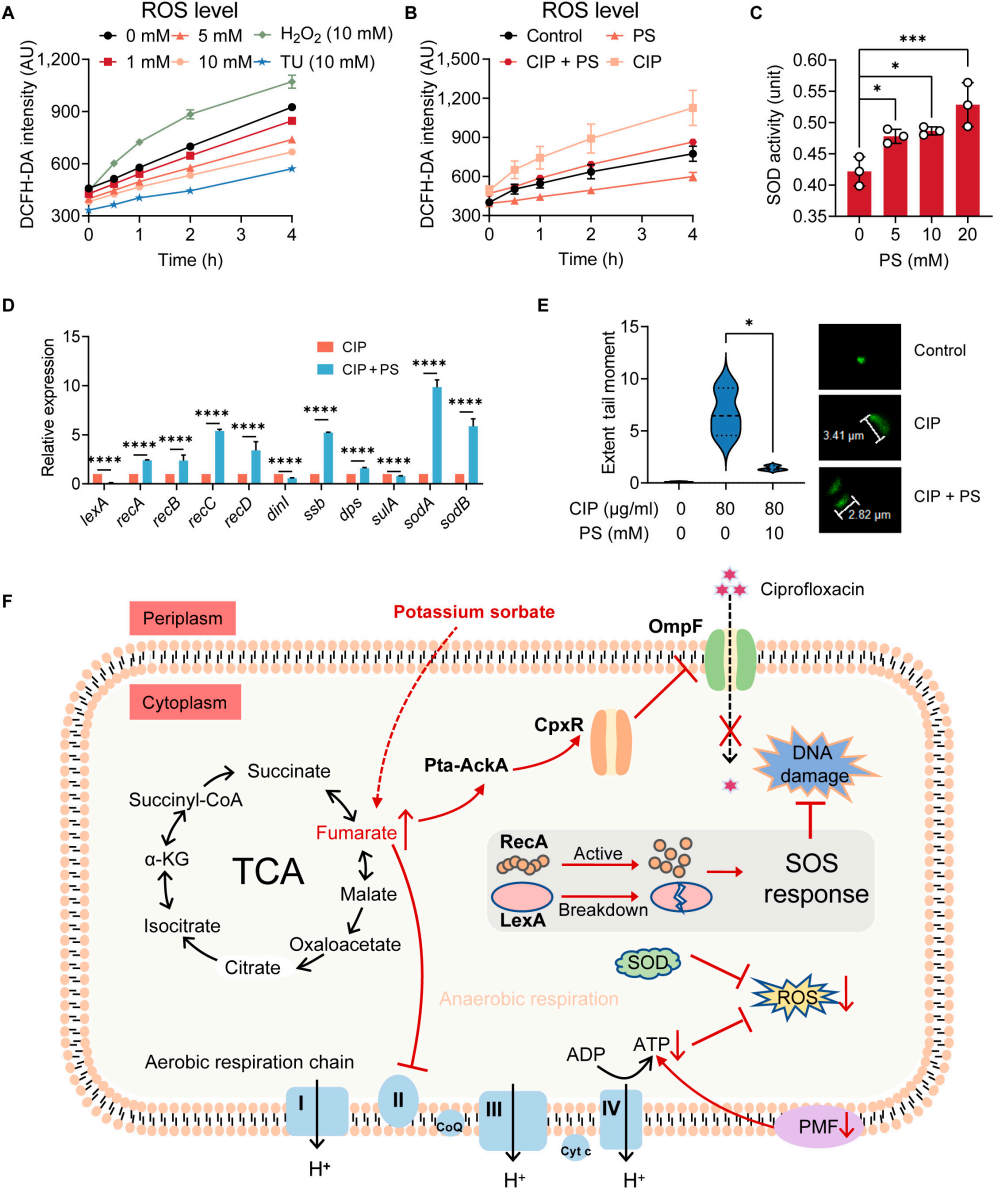
PS alleviates bacterial DNA damage induced by ciprofloxacin. (A and B) The level of ROS generated in *E. coli* G92 after incubated with PS (ranging from 0 to 10 mM, [A]), ciprofloxacin (80 μg/ml), or together with PS (10 mM, [B]) within 4 h, determined using DCFH-DA. H_2_O_2_ (10 mM) was used as a positive control, whereas thiourea (10 mM), a ROS scavenger, was used as a negative control. (C) The activity changes of SOD in *E. coli* G92 after 4 h of exposure to different concentrations of PS. (D) RT-qPCR analysis for the mRNA expression of SOS response genes (*recA*, *recB*, *recC*, *recD*, *dinI*, and *ssb*), oxidative stress gene (*dps*), genes controlling cell division (*sulA*), and genes encoding SOD (*sodA* and *sodB*) in *E. coli* G92 after cultured with ciprofloxacin (16 μg/ml) with or without PS (10 mM) for a period of 4 h. (E) Comet assay of DNA extracted from *E. coli* G92 after incubation with ciprofloxacin (80 μg/ml) alone or in combination with PS (10 mM). (F) A schematic diagram illustrating how PS protects bacteria from antibiotic killing. The data were shown as means ± SD, and the statistical significance was determined using a nonparametric one-way ANOVA. The levels of significance were denoted as *P < 0.05, ***P < 0.001, ****P < 0.0001. ADP, adenosine diphosphate.

Hydroxyl radical formation is an essential for bactericidal antibiotic-mediated killing [38]. The reduction in metabolic flux leads to a decrease in the generation of reactive metabolic byproducts like ROS. Accumulating evidence suggests that ROS formation is important in bactericidal antibiotic-mediated killing [39]. Therefore, we used 2′,7′-dichlorofluorescein diacetate (DCFH-DA) to label bacteria to explore the effect of PS or fumarate on ROS generation. The results showed that PS and fumarate remarkably inhibited the production of ROS in *E. coli* G92 (Fig. 6A and S4A) and PS antagonized the oxidative damage induced by ciprofloxacin (Fig. 6B). Previous transcriptome analysis showed that genes were partially enriched in oxidoreductase activity, so we determined the activity of superoxide dismutase (SOD). SOD can disproportionate superoxide anion radicals into hydrogen peroxide and oxygen, thereby maintaining intracellular ROS homeostasis [40]. The results showed that PS dose-dependently enhanced the activity of SOD (Fig. 6C). Consistently, the expression levels of SOD-related genes, such as *sodA* and *sodB*, were both increased by PS (Fig. 6D).

Given that the generation of ROS could provoke DNA damage and trigger the DNA damage response in cells [41], we speculated that the addition of PS induces the initiation of the DNA damage repair system, where DNA damage activates RecA, enhances the autocleavage of the LexA, and stimulates the SOS response. As expected, RT-qPCR analysis demonstrated that the expression levels of SOS response-related genes (*recB*, *recC*, *recD*, *dinI*, and *ssb*) were significantly enhanced after PS treatment than ciprofloxacin alone (Fig. 6D). Meanwhile, *dps*, which protects DNA against oxidative stress and reduces hydroxyl radical production, was significantly increased, coupled with the decrease in the expression of cell division genes (*sulA*), demonstrating that PS decreased DNA damage and leads to less need for inhibition of cell division. Furthermore, we conducted a comet experiment to detect the breakage in DNA. Visualized by fluorescent microscopy, there is a comet exhibiting a clear head consisting of undamaged DNA and a tail containing degraded fragments of DNA strands. The extent of DNA damage can be assessed by quantifying the displacement between the genetic material of the head and the resulting tail [42]. From the results, we found that ciprofloxacin increased the tail length while PS could shorten the tail length (Fig. 6E), suggesting that PS reduced the DNA damage caused by ciprofloxacin. Taken together, we conclude that PS-induced ciprofloxacin tolerance is mainly due to the inhibition of OmpF protein expression by the accumulation of fumarate, which reduces the uptake of ciprofloxacin. Moreover, increased fumarate inhibits aerobic respiration, reduces bacterial metabolism, and activates antioxidant responses, thus antagonizing bactericidal effect of antibiotics (Fig. 6F).

### PS confers ciprofloxacin tolerance in vivo

**Fig. 7.**
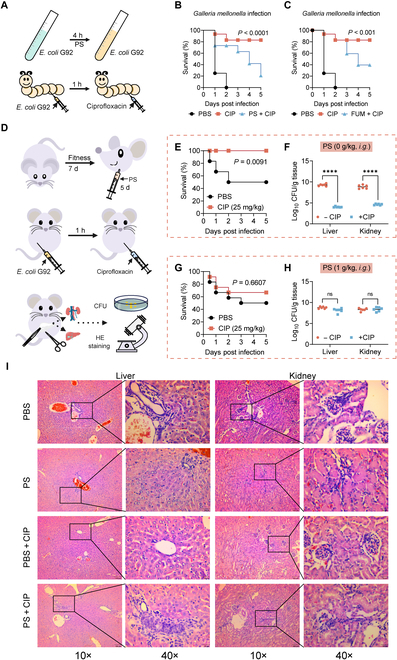
PS administration reduces ciprofloxacin effectiveness in vivo. (A) Experimental protocols for the infection model of *G. mellonella* larvae. (B and C) Survival rates of the *G. mellonella* larvae (*n* = 8 per group) infected by *E. coli* G92 (1.0 × 10^6^ CFUs) cocultured with PS (10 mM, [C]) or fumarate (50 mM, [D]) for 4 h and then treated with a single dose of ciprofloxacin (40 mg/kg). The statistical significance was determined by log-rank (Mantel–Cox) test. (D) Scheme of the experimental protocols for mouse peritonitis–sepsis infection. (E and G) Survival rates of the female BALB/c mice (*n* = 12 per group) infected intraperitoneally by a lethal dose of *E. coli* G92 (5.0 × 10^8^ CFUs), and then treated with PBS or ciprofloxacin (25 mg/kg). Mice were preadministrated with PBS (E) or PS (1 g/kg, [G]) for 5 consecutive days. The statistical significance was determined by log-rank (Mantel–Cox) test. (F and H) Bacterial loads in mice liver and kidney (*n* = 6 per group). Mice were preadministrated with PBS (F) or PS (1 g/kg, [H]) prior to infection as described above. The statistical significance was determined by Mann–Whitney U test. ****P < 0.0001. ns, not significant. (I) HE staining of mice liver and kidney under different treatments. A large amount of inflammatory cell infiltration was observed in PBS, PS, and PS plus CIP groups, indicating the decreased efficacy of CIP treatment in PS-preadministrated mice.

After demonstrating the role of PS in promoting antibiotic tolerance in vitro, we next investigated whether PS could induce bacterial tolerance in vivo and consequently diminish the therapeutic effectiveness of antibiotics. To evaluate this, we employed the *Galleria mellonella* infection models to assess the efficacy of ciprofloxacin (Fig. 7A). Insect larvae were infected with *E*. *coli* G92 without or with PS (10 mM) or fumarate (50 mM), followed by treatment with a single dose of ciprofloxacin (40 mg/kg). Consequently, bacteria cocultured with PS or fumarate were less susceptible to ciprofloxacin treatment, leading to a significantly reduced survival rate of larvae (Fig. 7B and C). Furthermore, a mouse peritonitis–sepsis model was applied to test the in vivo efficacy of ciprofloxacin (Fig. 7D). Prior to infection, the mice were orally administered with phosphate-buffered saline (PBS) or PS (1 g/kg) for a consecutive period of 5 d. All mice were then infected intraperitoneally with a lethal dose of *E. coli* G92 (5.0 × 10^8^ CFUs) and subsequently treated with ciprofloxacin (25 mg/kg). Consequently, a single dose of ciprofloxacin treatment drastically increased the 5-d survival rate of mice (*P* = 0.0091, Fig. 7E), and decreased the bacterial loads in the liver and kidney (Fig. 7F). In contrast, there was no change in mouse survival (*P* = 0.607, Fig. 7G) and bacterial loads (Fig. 7H) in the PS preadministered groups. Meanwhile, we performed hematoxylin and eosin (HE) staining to evaluate the pathological changes of liver and kidney. In both the liver and the kidney, the pathological lesions, such as inflammatory cell infiltration in the PBS and PS group, were comparable, while they disappeared in the ciprofloxacin treatment group. Also, the PS pretreated group displayed similar inflammation changes with PBS group (Fig. 7D), implying that PS also mediates antibiotic tolerance in mouse infection models. Collectively, these results indicate that PS impairs the therapeutic effect of ciprofloxacin in animal models of infection.

## Discussion

Unlike antibiotic resistance, which can be quantified by MIC test, antibiotic tolerance is still poorly characterized and often overlooked due to the paucity of quantitative indicators [43]. Moreover, the complex and detailed molecular mechanisms by which bacteria achieve tolerance remain poorly understood. Limited evidence showed that antibiotic tolerance can be acquired through genetic mutations or exposure to specific environmental conditions such as a poor nutritional environment [44]. Compounds closely related to human life sometimes exert selective pressure on bacteria in the environment, thereby affecting the activity of antibacterial therapeutic drugs. It has been shown that triclosan, a common disinfectant, induced in vivo tolerance to ciprofloxacin in *E. coli* in a murine model of urinary tract infection [45]. A recent research demonstrated that nitrite used in food preservation modulated bacterial tolerance to aminoglycoside antibiotics by targeting cytochrome heme–copper oxidases [46]. Therefore, a better understanding of the external conditions contributing to the formation of tolerance is particularly important in the fight against antibiotic-refractory bacteria.

In this study, we found that PS, commonly used as a bacteriostat and fungicide in various processed foods [47], triggers a high level of fluoroquinolone tolerance in *mcr*-positive bacteria. MCR protein is a member of the phosphoethanolamine transferase family and mediates colistin resistance [3]. Interestingly, this phenomenon was not detected in other kinds of drug-resistant bacteria, including *bla*_NDM-5_, *tet*(X4)- and *tmexCD1-toprJ1*-positive bacteria, and in *mcr*-negative bacteria (Fig. S10). Previous studies showed that *mcr* expression can affect the bacterial cell membrane permeability and functions [48,49]. Our results indicated that, following exposure to escalating levels of PS, the membrane permeability in *mcr*-positive and negative bacteria displayed a similar trend, while the variation tendency of ΔΨ was opposite (Fig. S11). Specifically, PS led to membrane depolarization in *mcr*-negative bacteria, whereas it resulted in membrane hyperpolarization in *mcr*-positive strain (Fig. S12). Our previous study showed that a food additive named sodium dehydroacetate induces tolerance to ciprofloxacin in *E. coli* by reducing bacterial PMF [50]. In addition, another study also pointed out that reduced PMF promotes bacterial tolerance to aminoglycosides [46]. In contrast, exogenous alanine or glucose restored the susceptibility of kanamycin-resistant *Edwardsiella tarda* to antibiotic killing by enhancing PMF [51]. Therefore, we speculated that the differential effects of PS on *mcr*-positive or negative bacteria may be related to opposite ΔΨ changes; however, the detailed reasons remain to be explored.

Our mechanistic studies demonstrated that PS-mediated tolerance is first initiated by inducing the accumulation of intracellular fumarate. Previous studies have shown that fumarate pre-exposure effectively reduced hydroxyl radical production even when cells were grown under aerobic conditions [30]. In agreement with these findings, we found that the addition of PS or fumarate reduced the oxygen consumption of bacteria and inhibited aerobic respiration. At the same time, fumarate activates the C4-dicarboxylates (C4DC) 2-component system DcuS–DcuR (DctA, DcuB, FumB, and FrdABC) [52], allowing bacteria to switch to anaerobic respiration and reduce ATP production. The potential difference inside and outside the membrane is a necessary factor for the generation of ATP, and the addition of PS reduces bacterial PMF, which further reduces ATP production and weakens bacterial metabolism. Consistently, previous studies showed that hypometabolism is a key factor in antibiotic tolerance formation [53,54]. Furthermore, fumarate as a kind of carbon source can activate CpxR transcription through Pta-AckA pathway [31]. The intermediate product acetyl phosphate promotes CpxR phosphorylation independent on CpxA [55], which leads to transcriptional repression of nonessential membrane protein complexes-related genes [56], including OmpF expression [33]. Consistently, we found that PS or fumarate can activate bacterial two-component system and repress *ompF* transcription. The dose-dependent down-regulation of OmpF protein greatly reduces the intracellular accumulation of drugs and ROS generation caused by antibiotics.

In addition, PS mitigated DNA damage mainly by activating SOS response. Quinolones cause bacterial DNA fragmentation [57], which is one of the mechanisms by which they kill bacteria, and they are potent inducers of SOS expression [58]. Activation of the SOS response promotes *gyrA* gene elevation after *E. coli* exposure to fluoroquinolones, thereby conferring resistance to this class of antimicrobials [59]. This is consistent with our findings that PS led to the overexpression of *gyrA*. We reasoned that the reduction in DNA damage under PS might be responsible for the specific tolerance to fluoroquinolones, rather than other antibiotics. Additionally, fumarase, a member of the DNA damage response in eukaryotic cells, can recruit to the nucleus upon DNA damage induction and protect cells from DNA double-strand break damage [60]. The absence of fumarase in cells can be replenished by high concentrations of fumarate, as fumarate boosts DNA repair by inhibiting the demethylation of histone H3 [61]. It was found that class-I fumarase genes *fumA* and *fumB* in *E. coli* also have similar effects [28]. The addition of PS clearly accumulated fumarate and enhanced the expression of *fumB*, which explained the attenuated DNA damage after coculture with PS.

Notably, our results also indicated that the addition of succinate, an intermediate of the TCA cycle, prevented PS-mediated antibiotic tolerance and restored ciprofloxacin activity against *mcr*-positive bacteria. This is mainly due to the increased expression of *SDH* and decreased expression of *FRD* after exposure to succinate, which is a pair of complementary systems [30]. The decreased *FRD* greatly impairs the utilization of increased fumarate by PS. Nevertheless, more clinical studies are still needed to demonstrate the potential of succinate in inhibiting PS-induced tolerance.

In summary, we reveal that a widely used food additive PS triggers a high level of fluoroquinolone tolerance in *mcr*-carrying bacteria both in vitro and in vivo by enhancing the intracellular accumulation of fumarate. Fumarate inhibits bacterial aerobic respiration, reduces OmpF expression, and promotes efflux pump, thereby decreasing antibiotic intracellular accumulation. Furthermore, fumarate reduces ROS generation and prevents antibiotic-induced DNA damage. Collectively, our findings illustrate a potential risk associated with the emergence of both colistin-resistant and fluoroquinolone-tolerant bacteria induced by PS in complex clinical settings.

## Materials and Methods

### Bacterial strains and culture

The bacterial strains utilized in this study are documented in Table S1. Unless otherwise stated, the bacteria were stored in aliquots with 20% glycerol at −80 °C and revived on Luria–Bertani (LB) agar, and cultured in Mueller–Hinton broth (MHB). All strains were grown at 37 °C with shaking at 200 rpm. Antibiotics were sourced from China Institute of Veterinary Drug Control, while other chemical reagents were purchased from Aladdin (Shanghai, China) or Sigma-Aldrich (Oakville, Ontario). Drugs were obtained as hydrochloride or sulfate, and all doses were calculated in the salt form. Drug stocks were prepared using sterile water and stored at −20 °C until use.

### MIC determination

MICs of antibiotics used in this study and PS were determined using serial 2-fold dilutions of drugs in MHB, and the samples were incubated for 18 h at 37 °C. The MIC values were determined as the lowest concentrations of antibiotics that prevent bacterial growth according to CLSI2021 guideline [62].

### Effect of sorbate on antibiotic killing

Bacteria carrying *mcr* gene were grown overnight and diluted 1:100 in 10 ml of fresh MHB medium containing various concentrations of sorbate (from 0 to 20 mM). Following incubation at 200 rpm for a duration of 4 h, bacterial cells were harvested and washed 3 times with PBS before being resuspended in M9CA medium (Sangon Biotech, Shanghai, China). Ciprofloxacin (20-fold MIC) was added for another 4 h. Then, 50 μl of bacterial solution was taken, serially diluted, and spot-plated on LB plates to enumerate the bacterial CFUs. The relative reduction in bacterial CFUs was calculated before and after the administration of the antibiotic.

The impact of PS on the effectiveness of fluoroquinolones (ciprofloxacin, levofloxacin, and norfloxacin, 40-fold MIC) and other bactericidal antibiotics (ampicillin, colistin, gentamicin, and tigecycline, 40-fold MIC) against *E. coli* B2 as mentioned above.

### TDtest assay

Filter paper was cut into circles with a diameter of 6 mm, and then they were sterilized and impregnated with either 5 μl of a 40% sterile glucose solution for glucose disks, or 5 μl of ciprofloxacin solution (5,120 μg/ml) for antibiotic disks. The impregnated disks were then left to dry at room temperature. *E. coli* B2 was cultivated overnight and diluted 1:100 into 10 ml of blank MHB or MHB containing PS (20 mM) for 4 h. Then, the bacteria were washed 3 times with PBS and diluted to 10^6^ CFU/ml. The bacteria were subsequently plated on an LB agar plate, with the antibiotic disk placed on top of the agar plate. The antibiotic disks were replaced with glucose disks after 18 h and incubated overnight. The zone of inhibition was checked for colonies.

### The minimum duration of killing determination

The minimum duration of killing (MDK) was determined by time-killing assay. Briefly, the bacteria cultures were incubated overnight and subsequently diluted 1/100 into 10 ml in fresh MHB with or without PS (10 mM) for 4 h. Then, the bacteria were washed 3 times with PBS and resuspended in M9CA, and ciprofloxacin (10-fold MIC) was added. At different time points (0, 2, 4, and 6 h), 50 μl of bacterial solution was taken, serially diluted, and spot-plated on LB plates for counting CFUs per milliliter. MDK_99_ was defined as the shortest time required to achieve a 99% reduction in total CFUs from the initial inoculum at a particular concentration.

### Transcriptomic analysis

*E. coli* G92 cultures were incubated overnight and subsequently diluted 1/100 into 10 ml in fresh MHB with or without PS (10 mM) for 4 h. Then, bacteria were harvested and washed with PBS for 3 times. TruSeqTM Stranded Total RNA Library Prep Kit reagents were used to construct the library. Briefly, total RNA was extracted through the EASYspin Plus kit, and the concentration and purity of the extracted RNA were assessed using NanoDrop 2000. The integrity of the RNA was evaluated through agarose gel electrophoresis, and the RNA integrity number was measured using the Agilent 2100 system. Finally, sequencing was performed using a 2*150-bp/300-bp protocol. The raw data after quality control were compared with the reference genome to obtain mapped data (reads) for subsequent analysis. Functional annotation was performed by comparing with GO and KEGG database. RSEM software was used to quantitatively analyze the expression levels of genes, and DESeq2 was used to identify DEGs.

### LC-MS/MS analysis

Overnight *E. coli* G92 were diluted 1:100 into fresh MHB or MHB supplemented with PS (10 mM), and then the bacteria were cultured on a shaker at 37 °C, 200 rpm for 4 h. Cells were collected and washed 3 times with PBS and then diluted into 10^5^ CFU/ml. Subsequently, the bacterial solution was supplemented with ciprofloxacin (16 μg/ml) and subjected to incubation at 37 °C with shaking at 200 rpm for 1 h.

#### Metabolite extraction

The pellets of bacteria were dissolved in 200 μl of water after centrifuging for 3 min at a speed of 12,000 rpm. The suspension was frozen and thawed 3 times in liquid nitrogen (3 min each time) and then treated in a water bath at 65 °C for another 3 min to lyse the sample. The resulting mixture was centrifuged at a speed of 12,000 rpm for 3 min, and the supernatant was collected. Remaining pellet was resuspended in 200 μl of methanol, followed by vortexing and centrifugation at 12,000 rpm for 3 min. Finally, the supernatants were combined, and 600 μl of acetonitrile was added and vortexed. The supernatant was filtered through a 0.22-μm needle filter in preparation for subsequent liquid chromatography-mass spectrometry analysis.

#### Liquid chromatography-mass spectrometry

Intracellular fumarate (114.7/32.1*) concentrations were quantified using a high-performance liquid chromatography system (Agilent 1260) coupled to a mass spectrometer (SCIEX 6500 QTRAP, Applied Biosystem) with negative electrospray ionization in multiple reaction monitoring mode [50]. Liquid chromatography separation conditions: mobile phase A (0.1% formic acid aqueous solution) and mobile phase B (acetonitrile solution); chromatographic column: C18 column (4.6 mm × 250 mm). The flow rate was set at 0.5 ml/min, and the gradient elution ratio was as follows: 0.1 to 2.0 min, 95% A; 2.0 to 5.0 min, 10% A; 5.0 to 8 min, 10% A; 8.0 to 8.1 min, 95% A; 8.1 to 10.0 min, 95% A; 10-μl injection volume.

Intracellular ciprofloxacin (331.34/231*) concentrations were determined using negative electrospray ionization in multiple reaction monitoring mode. Liquid chromatography separation conditions: mobile phase A (10 mM ammonium acetate) and mobile phase B (acetonitrile solution); chromatographic column: C18 column (4.6 mm × 250 mm). The flow rate was set at 0.5 ml/min, and the gradient elution ratio was as follows: 0.1 to 2.0 min, 95% A; 2.0 to 5.0 min, 10% A; 5.0 to 8 min, 10% A; 8.0 to 8.1 min, 95% A; 8.1 to 10.0 min, 95% A; 10-μl injection volume.

### Biochemical parameter assay

Protocols for pretreatment of cells are listed below. Overnight, *E. coli* G92 was diluted 100-fold into the blank MHB and cultivated at 37 °C and 200 rpm for 4 h. After cells were collected, they were washed with PBS 3 times and resuspended with M9CA. Fluorescent dyes were introduced and subjected to incubation at a temperature of 37 °C for a duration of 30 min. Subsequently, probe-labeled cells (190 μl) were added into a 96-well plate. Different concentrations of PS (0, 1, 5, and 10 mM) or ciprofloxacin (0, 8, 80, and 160 μg/ml) were added alone or used together (10 mM PS with different concentrations of ciprofloxacin). Using a microplate reader (Infinite E Plex), fluorescence intensity was measured after 1 h of incubation at 37 °C in the dark.

#### Bacterial respiration assay

Resazurin [63] (Aladdin) was dissolved in water and prepared as a sterile stock solution of 10 μg/ml. Bacterial cells, in different concentrations of PS or ciprofloxacin or in combination, were added into a 96-well plate, and resazurin solution (0.1 μg/ml) was added. Fluorescence intensity was immediately assessed at an excitation wavelength of 550 nm and an emission wavelength of 590 nm within a duration of 30 min.

Oxygen consumption was determined using Oxygen Consumption Rate Assay Kit (BBoxiProbe) [64]. Cells were collected and resuspended with MHB to an optical density at 600 nm (OD_600_) of 0.5. Fluorescence intensity was assessed at 603 nm with an excitation wavelength of 468 nm.

#### Membrane depolarization assay

Fluorescent dye DiSC_3_(5) [50] (Aladdin) at a final concentration of 0.5 μM was used to measure membrane potential change. Fluorescence intensity was measured at 670 nm with an excitation wavelength of 622 nm.

#### Total ROS measurement

Fluorescent dye DCFH-DA (10 μM, Beyotime) [65] was used to determine the production of ROS in *E. coli* G92. The labeled bacteria were harvested and washed 3 times with PBS and cocultured with PS, ciprofloxacin, or their combination. Fluorescence intensity was measured at 525 nm with the excitation wavelength at 488 nm. H_2_O_2_ (10 mM) was used as a positive control, while thiourea (10 mM) was used as a negative control.

#### Measurement of intracellular ATP levels

The ATP levels of *E. coli* G92 were quantified utilizing an Enhanced ATP Assay Kit (Beyotime). After pretreatment as described above, bacteria were centrifuged, washed, and resuspended in lysis buffer. After lysis, the removal of bacterial debris was achieved through centrifugation at a speed of 12,000 rpm for a duration of 5 min at a temperature of 4 °C, and the supernatant was taken as a sample for later use. Subsequently, 100 μl of ATP detection working solution was introduced into a 96-well plate and placed for 5 min at room temperature. Following this, 20 μl of the aforementioned sample was added and mixed them evenly. The relative light unit value was measured by an Infinite E Plex Microplate reader (TECAN) after an interval of 5 s. The concentration of ATP in the samples was subsequently determined by referencing the standard curves.

#### SOD activity determination

By using the Total Superoxide Dismutase Assay Kit with WST-8 (Beyotime, China) [66], *E. coli* G92 was tested for intracellular SOD activity after treatment with PS at various concentrations.

#### SDH activity determination

Intracellular SDH activity of *E. coli* G92 cocultured by various concentrations of PS (0 to 20 mM) or fumarate (0 to 100 mM) or cocultured with succinate (10 mM) was detected by Succinate Dehydrogenase (SDH) Kit (Grace Biotechnology, China).

#### Outer membrane permeability assay

Fluorescent dye *N*-phenyl-1-naphthylamine [67] at a final concentration of 10 μM was used to measure outer membrane permeability. Fluorescence units were monitored at 420 nm with the excitation wavelength at 350 nm.

#### Cell membrane integrity assay

Fluorescent probe propidium iodide [68,69] at a final concentration of 5 μM was used to detect the integrity of cell membrane. Fluorescence units were measured at 535 nm with the excitation wavelength at 615 nm.

#### Efflux pump assay

A fluorescent probe ethidium bromide (EtBr) [70] at a final concentration of 5 μM was used to evaluate the function of efflux pump. Fluorescence units were monitored at 600 nm with the excitation wavelength at 530 nm within 30 min.

### Flow cytometry analysis

Bacterial PMF was determined using DiOC_2_(3) [71]. *E. coli* G92 was cocultured with PS (0, 5, 10, and 20 mM) or fumarate (0, 25, 50, and 100 mM) at 37 °C for 4 h. Cells were harvested and washed, and resuspended with M9CA to OD_600_ = 0.3. DiOC_2_(3) was dissolved in dimethyl sulfoxide and prepared as a sterile stock solution of 3 mM. During oscillation at 37 °C for 15 min, the bacteria were incubated with 20 μl of DiOC_2_(3). Dye-free samples were used to control for autofluorescence and carbonyl cyanide 3-chlorophenylhydrazone (5 μM) was added as the depolarized control sample. The detection of green fluorescence was performed using a band-pass filter with a bandwidth of 488 to 530 nm, whereas red fluorescence was detected using a band-pass filter with a bandwidth of 488 to 610 nm. The CytExpert Flow Cytometer (Beckman, USA) determined a total of 100,000 ungated events in each sample. Then, they were analyzed by FlowJo 10.8.1 software (Becton, Dickinson and Company, USA). The calculation of PMF was based on the intensity ratio of red/green fluorescence.

### Construction of *E. coli* knockout mutants

*FRD*, *PTA-ackA*, and *OmpF* were knocked out using the CRISPR/Cas9 system [72]. Briefly, plasmid pHCY-25A was transferred into *E. coli* J53 and prepared into chemically competent cells. Plasmid pGS150A (or pGS150B; 5 μl) was converted into *E. coli* J53 (pHCY-25A or pGS150B) and resuscitated for 45 min. Then, cells were seeded in LB agar plates containing kanamycin and ampicillin and cultured overnight at 30 °C. After primary selection, the bacteria were inoculated into LB broth (containing kanamycin and ampicillin) and cultured at 30 °C for 2 h. Isopropyl β-D-thiogalactopyranoside ( was added into an LB tube, and the mixture was cultured at 30 °C for 1 h; then, L-arabinose was added and incubated at 30 °C for 3 h. Bacterial culture was diluted 1:100 into fresh LB, and then cells were seeded in LB agar plates containing kanamycin, ampicillin, and L-arabinose. A single colony was selected for PCR validation. Then, bacteria were cultured on LB plates (containing sucrose) at 37 °C to eliminate plasmid. Knockout mutants were confirmed by colony PCR with optimized primers.

### Gel electrophoresis and immunoblotting assays

Overnight *E. coli* G92 were diluted 1:100 into fresh MHB with PS (0 to 20 mM) or fumarate (0 to 100 mM) at 37 °C for 4 h. Bacteria were harvested and washed, and resuspended with PBS containing lysozyme (1 mg/ml). Ultrasound was used to break up the bacteria. Protein concentration was detected using BCA Protein Assay Kit (Beyotime). SDS-PAGE Sample Loading Buffer (6X, Beyotime) was added and boiled for 10 min to denature. One-Step PAGE Gel Fast Preparation Kit (10%) (Vazyme Biotech, Nanjing, China) was used to make resolving gel and stacking gel. Until the dye front reached the bottom of the gel, electrophoresis was performed at a constant voltage of 150 V. For Western blot analysis, the proteins in the gel were transferred to nitrocellulose filter (NC) membranes in transfer buffer at 200 mA for 90 min and then were blocked through NcmBlot blocking buffer (NCM Biotech) at 37 °C for 15 min. NC membranes underwent 3-times washes with tris-buffered saline with Tween 20 (TBST) buffer for a duration of 10 min each. Subsequently, the NC membranes were incubated subjected at 4 °C with rabbit antiserum targeting *E. coli* OmpF at a dilution of 1:1,000 in primary antibody dilution buffer. Following 3 rinses of 10 min each with TBST buffer, the NC membranes were exposed to goat anti-rabbit immunoglobulin G-horseradish peroxidase at a dilution of 1:3,500 in TBST containing 5% skim milk for 2 h at 37 °C. The bands were detected with enhanced chemiluminescence solution using BeyoECL Moon Kit (Beyotime) and visualized using LAS4000 (GE Healthcare). The gray value was calculated by ImageJ. Protein bands were visualized with Coomassie Blue Fast Staining Solution (Beyotime).

### RT-qPCR analysis

*E. coli* G92 was cultured until reaching the stationary phase and diluted at a ratio of 1:100 into 2 ml of fresh MHB for 4 h before the bacterial cells were harvested and washed 3 times with PBS. Then, the bacterial were cultured with ciprofloxacin (16 μg/ml) with or without PS (10 mM) for a period of 4 h. Subsequently, the total RNA was extracted by Bacteria RNA Extraction Kit (Vazyme) and quantified by determining the absorbance ratio (260 nm/280 nm). The total RNA was reverse-transcribed using PrimeScript RT Kit and gDNA Eraser (Takara, Dalian, China), following the provided instructions. RT-qPCR analysis was performed by 7500 Fast Real-Time PCR System (Applied Biosystem, CA, USA) using the ChamQ SYBR Color qPCR Master Mix (Vazyme) with the primers (Table S6). A 2-step PCR amplification standard procedure was used for thermal cycling (40 cycles of 95 °C for 30 s, 95 °C for 10 s, and 60 °C for 30 s). Based on the 2^−ΔΔCt^ method, the fold change of mRNA expression relative to a reference gene (16S ribosomal RNA) was calculated.

### Comet assay

OxiSelect Comet Assay Kit (Cell Biolabs, USA) was used to assess DNA damage. Briefly, comet agarose was subjected to a temperature of 95 °C in a water bath until it reached a liquid state. *E. coli* G92 was cultivated with ciprofloxacin (80 μg/ml) or in combination with PS (10 mM) at 37 °C for 4 h. Cell samples (10^5^ CFU/ml) were in combination with comet agarose at a ratio of 1:10 (v/v), thoroughly mixed, and promptly applied onto the upper layer of the Comet agarose base. The resulting slide was then transferred to a small container containing 25 ml of lysis buffer and incubated for a period of 60 min in a light-restricted environment. Subsequently, the lysis buffer was substituted with 25 ml of prechilled alkaline solution. The slides were transferred horizontally into the horizontal electrophoresis tank and electrophoresed with 27 V, 300 mA for 20 min. Then, the slides were submerged in prechilled deionized H_2_O for a duration of 2 min, followed by aspiration and repetition of this process 2 more times. The final rinse water was aspirated and substituted with cold 70% ethanol for 5 min before air-dried. Finally, 100 μl per well of diluted Vista Green DNA dye was introduced. The slides were observed by Leica TCS SP8 STED confocal laser microscopy (Leica, Germany) using fluorescein isothiocyanate filters.

### Animal experiments

Female BALB/c mice (aged 6 to 8 wk) were procured from the Comparative Medicine Centre of Yangzhou University (Jiangsu, China). Mice were acclimatized for 1 week before being infected. Animal experiments were conducted in compliance with the applicable guidelines and regulations set forth by Jiangsu Laboratory Animal Welfare and Ethical of Jiangsu Administrative Committee of Laboratory Animals (ID: SYXK2022-0044). The use of experimental animals was authorized by the Jiangsu Association for Science and Technology under license number SCXK-2022-0009.

#### Mouse peritonitis–sepsis infection model

A total of 48 female BALB/c mice were intraperitoneally injected with PBS or 1 g/kg PS for 5 d and then intraperitoneally infected with *E. coli* G92 suspension at a dose of 5.0 × 10^8^ CFUs. After 1 h of infection, the mice were randomly divided into control and antibiotic treatment groups, with 12 mice in each group. The control group received a single intraperitoneal dose of PBS, while the antibiotic treatment group received ciprofloxacin at a dosage of 25 mg/kg. The survival of the mice was monitored for a period of 5 d. The liver and kidney were aseptically removed and separated into 2 parts for CFU estimation or hematoxylin and eosin staining.

#### *Galleria mellonella* infection model

*Galleria mellonella* larvae obtained from Huiyude Biotech (Tianjin, China) were randomly assigned to 6 groups with 8 larvae in each group. Then, they were infected with *E. coli* G92 suspension (10 μl, 1.0 × 10^6^ CFUs per larva, 4 groups) or *E. coli* G92 suspension (10 μl, 1.0 × 10^6^ CFUs per larva) precultured with PS (10 mM) or fumarate (50 mM), respectively. After 1 h of infection, *Galleria mellonella* larvae were treated with PBS or ciprofloxacin (40 mg/kg). Survival rates of *Galleria mellonella* larvae were recorded for a period of 5 d.

### Statistical analyses

Statistical analysis was conducted using GraphPad Prism Version 9.0. All data were obtained from a minimum of 3 biological replicates. For the in vitro studies, the statistical significance was determined through an unpaired *t* test between 2 groups or one-way analysis of variance (ANOVA) among multiple groups. For the in vivo studies, log-rank (Mantel–Cox) test or the Mann–Whitney U test was used to calculate *P* values. Significance levels are indicated by asterisks as follows: ns, not significant; *P < 0.05, **P < 0.01, ***P < 0.001, ****P < 0.0001.

## Data Availability

RNA-sequencing data have been deposited in the National Center for Biotechnology Information Sequence Read Archive database (PRJNA1011224).
