# Supplementary material for: A Dietary Source of High Level of Fluoroquinolone Tolerance in mcr-Carrying Gram-Negative Bacteria
Source: Research (Wash D C). 2023 Oct 6;6:0245. doi: 10.34133/research.0245 (PMC10557118; doi:10.34133/research.0245)
Supplement: Supplementary 1 — Figs. S1 to S12 Tables S1 to S7 [file research.0245.f1.docx]

**Supplemental Material**

**Figures**


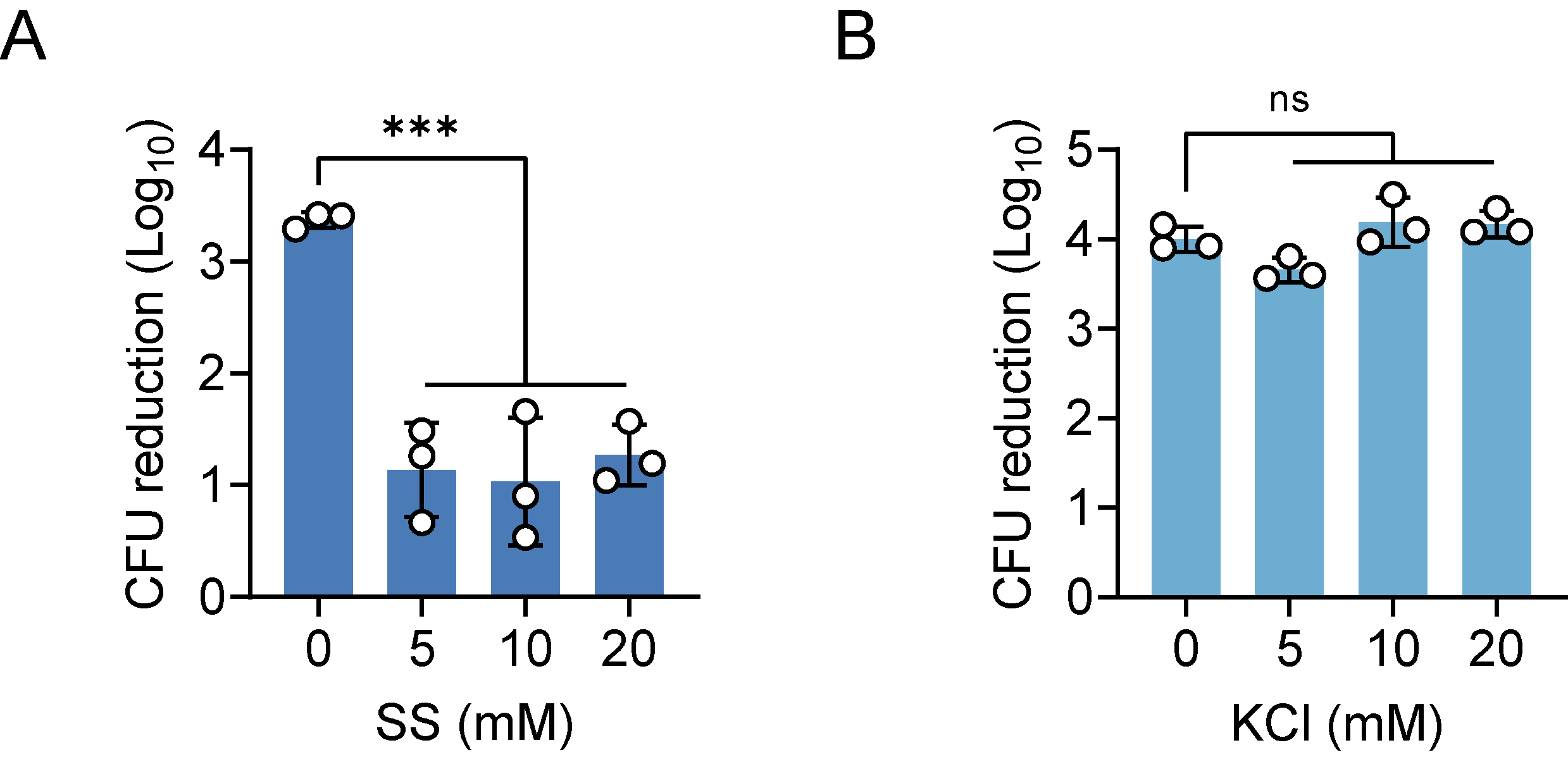


**Figure S1. Co-cultivation of *E. coli* G92 with sodium sorbate weakens its susceptibility to ciprofloxacin.**

CFU reduction (Log_10_) of *E. coli* G92 cultured with sodium sorbate (ranging from 0 to 20 mM, **A**) or potassium chloride (ranging from 0 to 20 mM, **B**) after treated with ciprofloxacin. All data from triplicate biological experiments were presented as means ± SD, and the significance determined by non-parametric one-way ANOVA. ns, not significant, ****P* < 0.001.


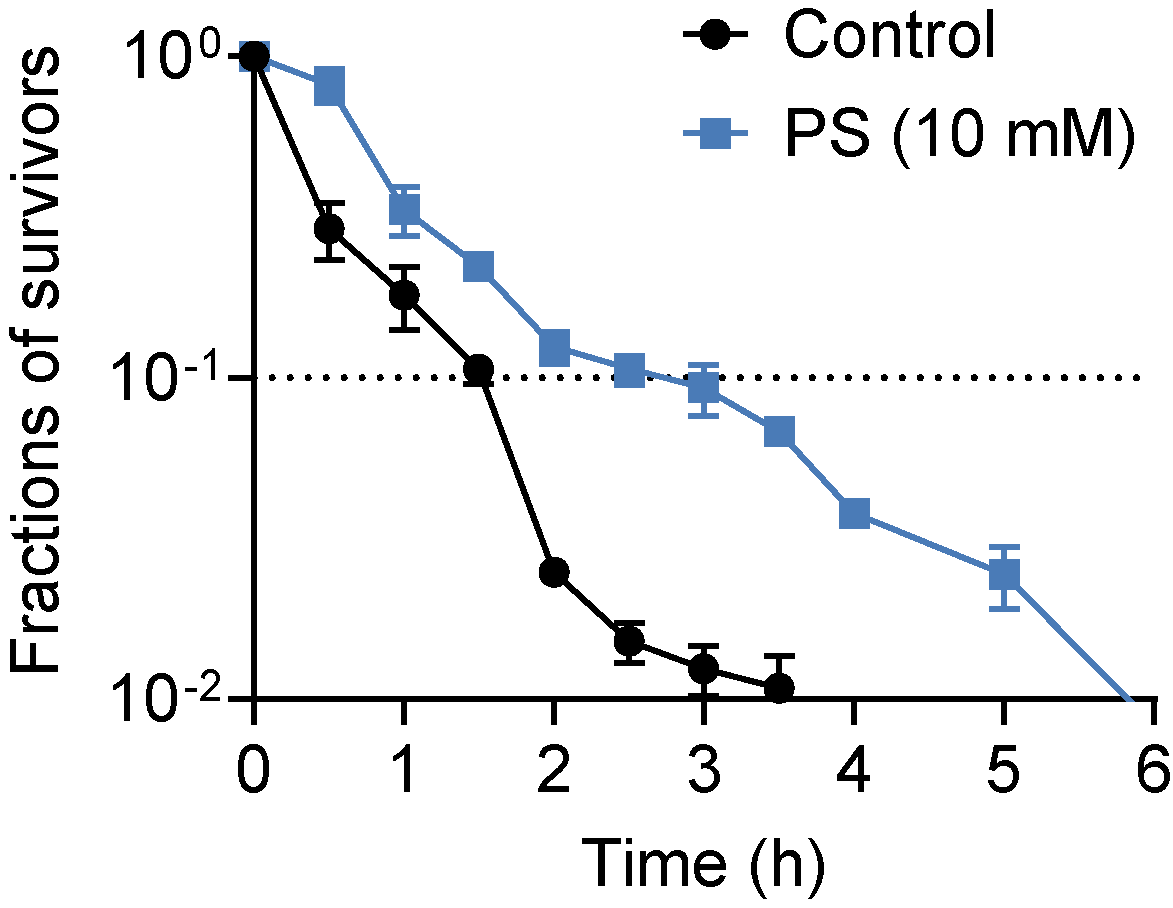


**Figure S2. The minimum duration for killing 99% (MDK_99_) for *E. coli* G92.**

Time-killing kinetics of *E. coli* G92 following exposure to ciprofloxacin (10-fold MIC) after co-cultured with PS (10 mM) for 4 h. Black horizontal dotted line shows minimum duration of killing times needed to kill 90% of the initial inoculum (MDK_90_). All data from triplicate biological experiments were presented as means ± SD.


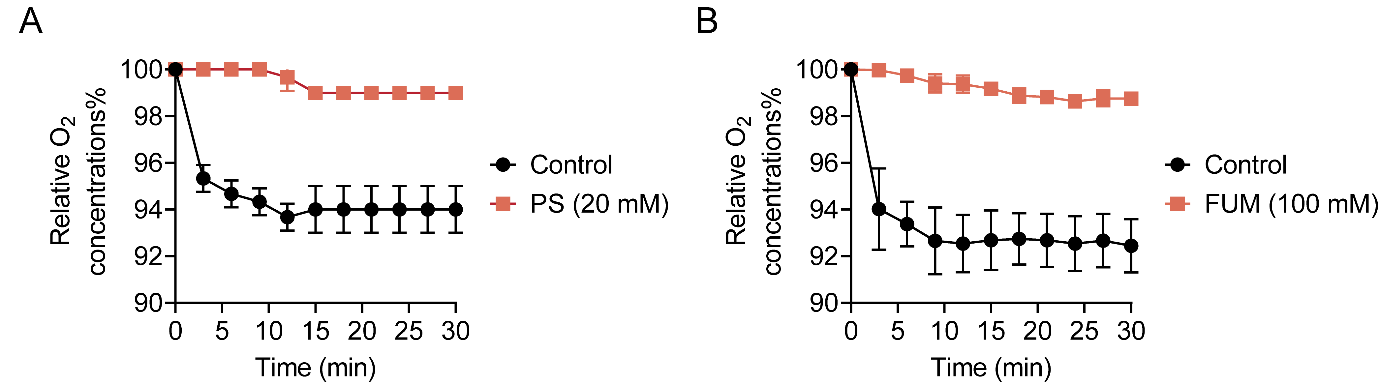


**Figure S3. The curves of oxygen consumption.**

Fluorescence of the oxygen probe in the cells was monitored within 30 min after the addition of PS (20 mM, **A**) or fumarate (100 mM, **B**). All data from triplicate biological replicates were expressed as mean ± SD.

**
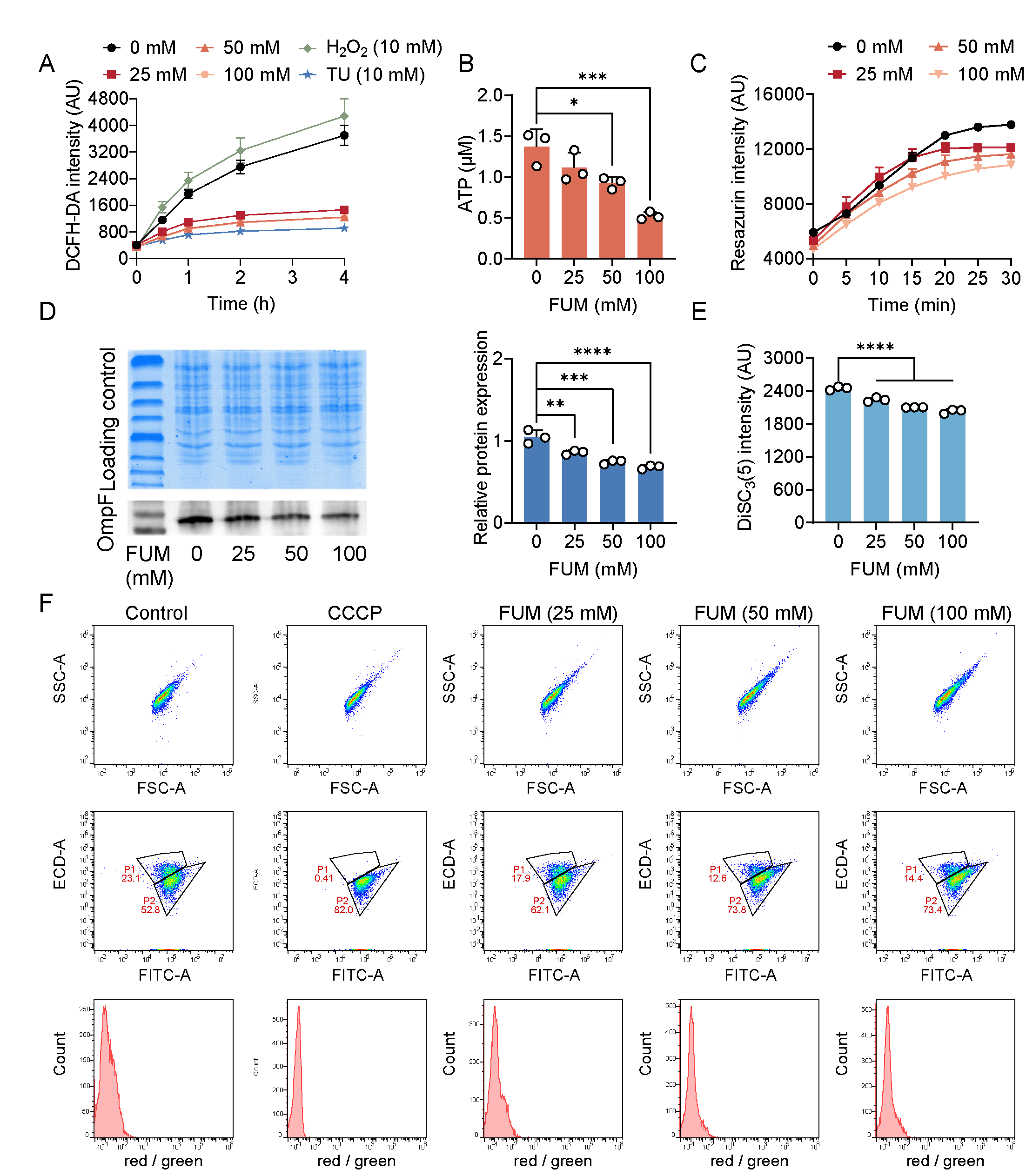
**

**Figure S4. Fumarate restrains bacterial metabolism**

**(A)** ROS production of *E. coli* G92 during incubation with fumarate (ranging from 0 to 100 mM) for 4 h, determined using 2′,7′-dichlorofluorescein diacetate (DCFH-DA). H_2_O_2_ (10 mM)-treated bacteria were used as a positive control, whereas thiourea (10 mM), a ROS scavenger, was used as a negative control.

**(B)** Intracellular ATP concentration after *E. coli* G92 pretreated by different concentrations of fumarate (ranging from 0 to 100 mM).

**(C)** Respiration of *E. coli* G92 during incubation with fumarate (ranging from 0 to 100 mM) during 30 min, determined using resazurin.

**(D)** *E. coli* G92 were co-cultured with fumarate (ranging from 0 to 100 mM) for 4 h and subjected to protein extraction and western blotting. The chart presents the relative amount of OmpF and normalized to Coomassie Brilliant Blue.

**(E)** Membrane potential changes induced by fumarate (ranging from 0 to 100 mM), determined by fluorescence dye DiSC_3_(5).

**(F)** The proton motive force (PMF) of *E. coli* G92 after co-cultured with fumarate, CCCP was used to reduce the proton gradient to destroy the PMF.

All data from triplicate biological replicates were expressed as mean ± SD, and the statistical significance was determined by one-way ANOVA, and shown as **P* < 0.05, ***P* < 0.01, ****P* < 0.001, *****P* < 0.0001.

**
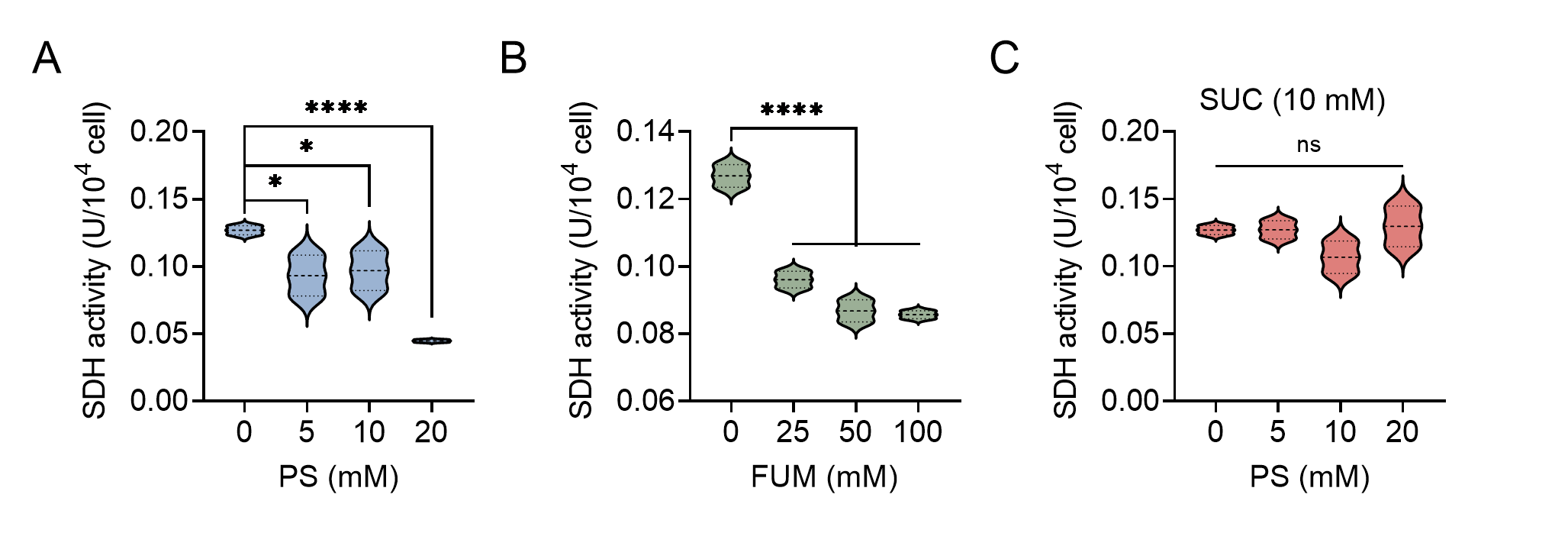
**

**Figure S5. Determination of succinate dehydrogenase (SDH) activity in *E. coli* G92**

The SDH activity (U/10^4^ cell) of *E. coli* G92 during incubation with PS (ranging from 0 to 20 mM, **A**) and fumarate (ranging from 0 to 100 mM, **B**) or co-cultured with PS and succinate (10 mM, **C**). All data from triplicate biological replicates were expressed as mean ± SD, and the statistical significance was determined by one-way ANOVA, and shown as **P* < 0.05, *****P* < 0.0001. ns, not significant.


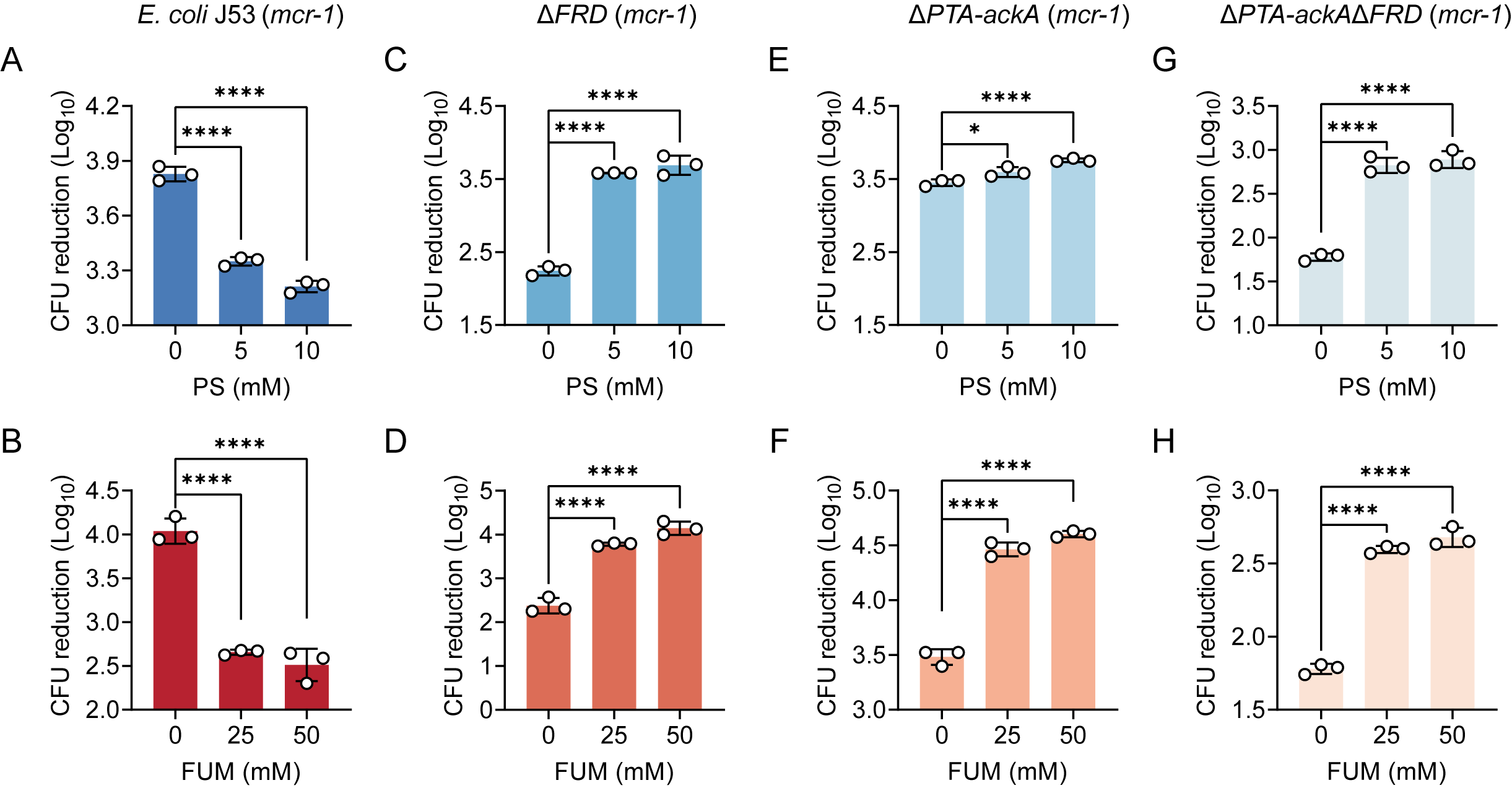


**Figure S6. PS or fumarate-mediated fluoroquinolones tolerance was reversed in FRD or PTA-ackA knockout strains.**

PS **(A)** and fumarate **(B)** dose-dependently decreased ciprofloxacin activity against *E. coli* J53 (*mcr-1*), whereas this phenomenon was abolished in Δ*FRD* (*mcr-1*) **(C and D)**, Δ*Pta-AckA* (*mcr-1*) **(E and F)** and the double knockout strain Δ*Pta-AckA*Δ*FRD* (*mcr-1*) **(G and H)***.*
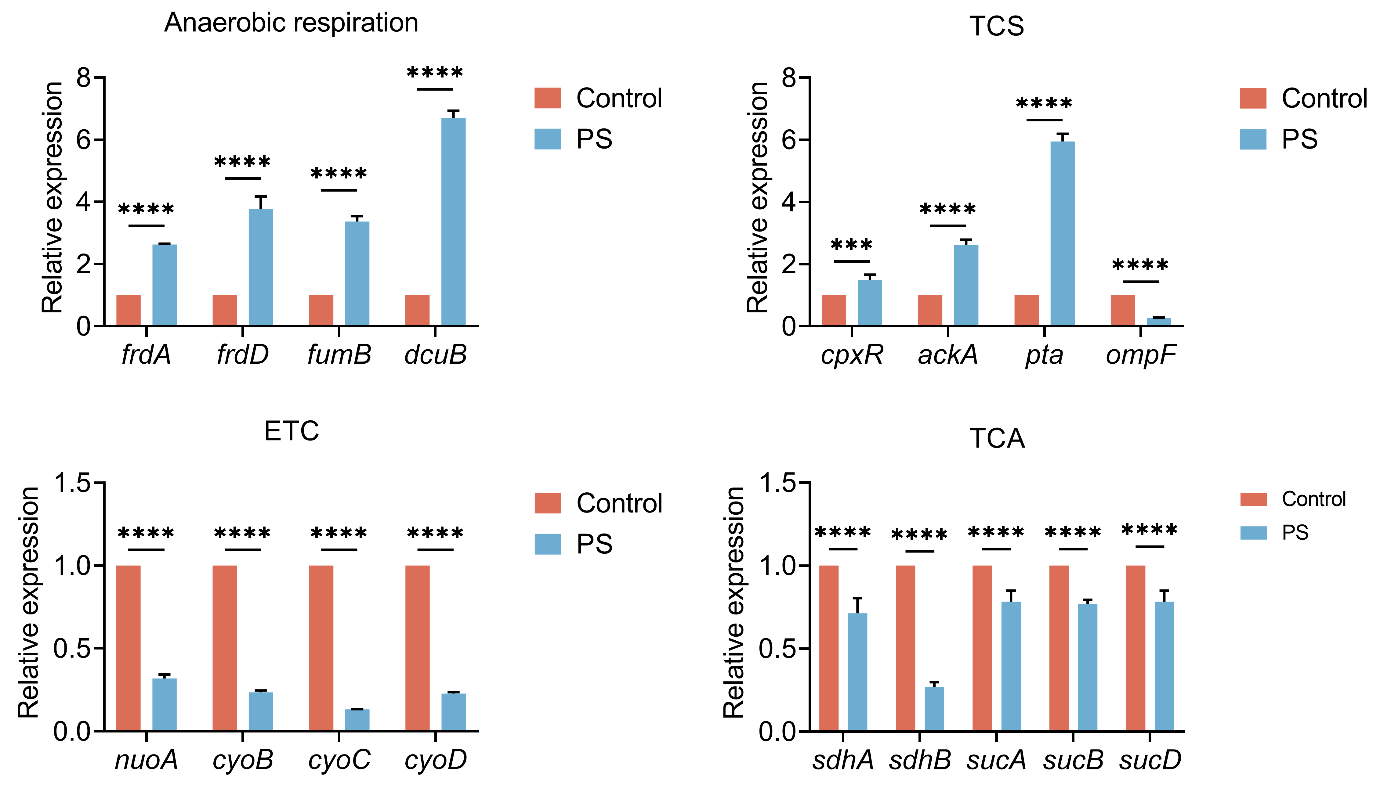


**Figure S7. Relative expression of representative genes in *E. coli* G92 after PS co-cultured for 4 h by RT-qPCR analysis.**

Transcription levels of several representative anaerobic-respiration-related genes (*frdA*, *frdD*, *fumB*, *dcuB*) and TCS-related genes (*cpxR*, *ackA*, *pta*) except *ompF*. Meanwhile, PS downregulates the transcription of ETC (*nuoA*, *cyoB*, *cyoC*, *cyoD*) and TCA-related genes (*sdhA*, *sdhB*, *sucA*, *sucB*, *sucD*) were detected. All data were presented as mean ± SD and the significance determined by unpaired *t* test (*****P* < 0.0001).


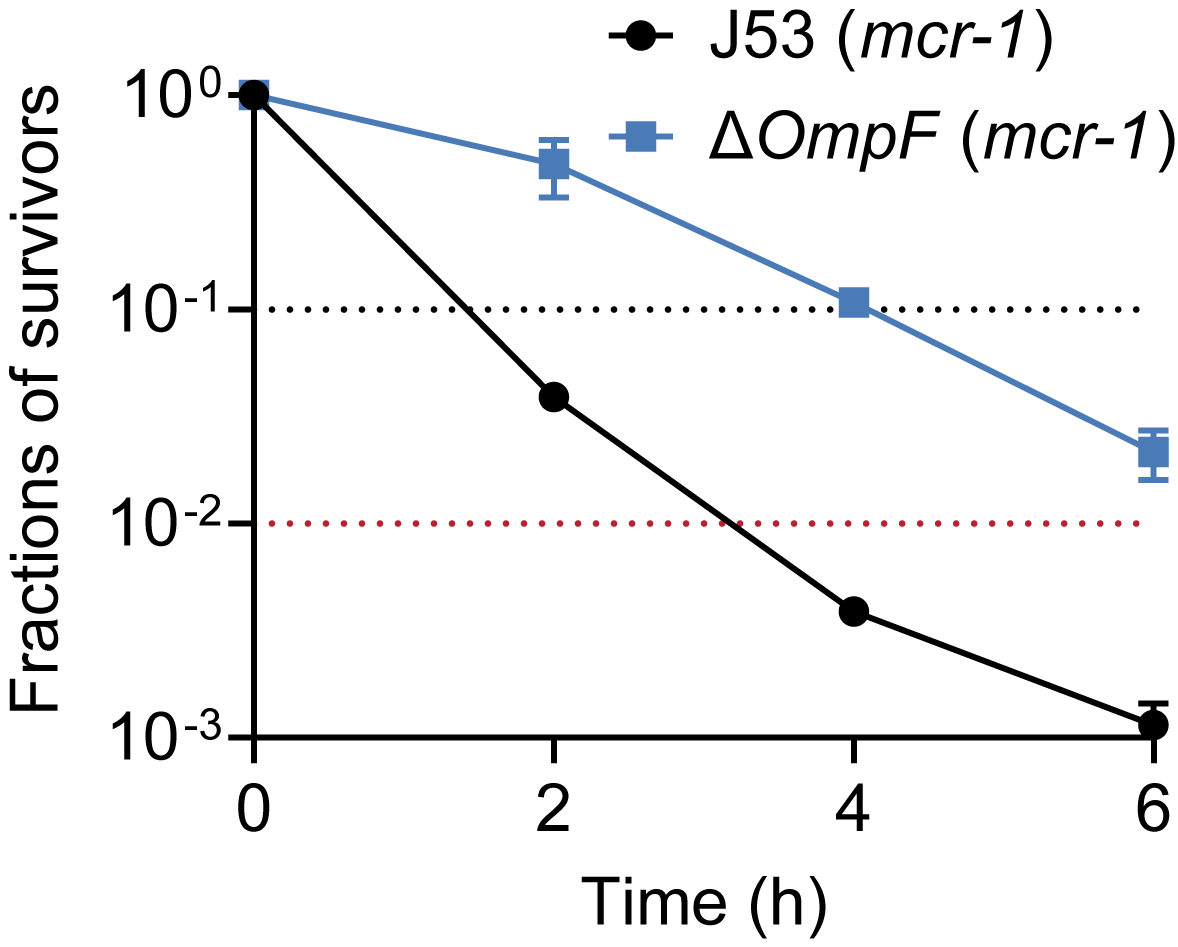


**Figure S8. The minimum duration for killing (MDK_99_) for *E. coli* J53 (*mcr-1*) and the knockout strain Δ*OmpF* (*mcr-1*).**

Time-killing kinetics of *E. coli* J53 (*mcr-1*) and Δ*OmpF* (*mcr-1*) following exposure to ciprofloxacin (10-fold MIC) after cultured for 4 h. Black horizontal dotted line shows minimum duration of killing times needed to kill 90% of the initial inoculum (MDK_90_), while red horizontal dotted line shows minimum duration of killing times needed to kill 99% of the initial inoculum (MDK_99_). All data from triplicate biological experiments were presented as means ± SD.


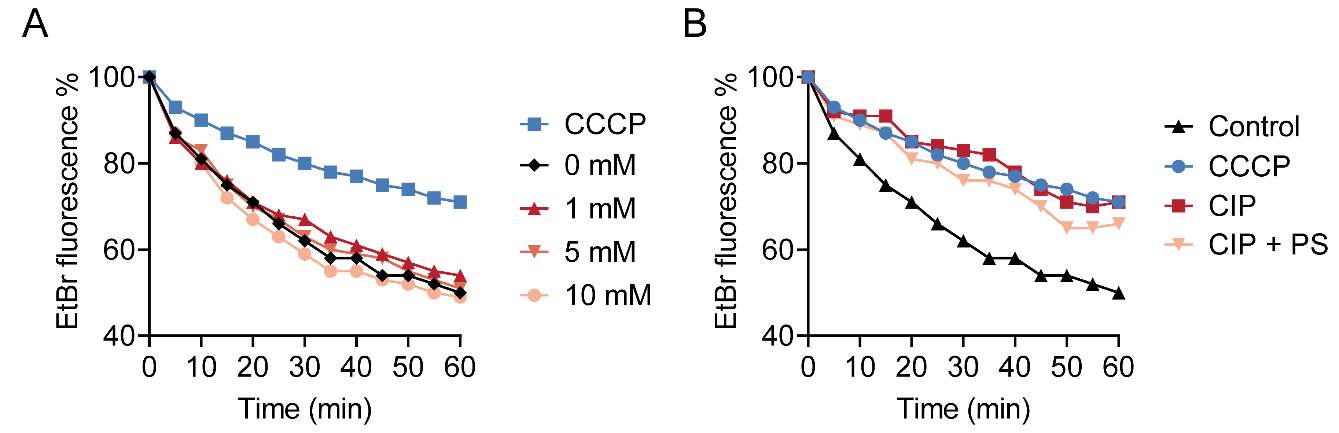


**Figure S9. PS** **enhances efflux pump function in a dose-dependent manner.**

The percentage of EtBr fluorescence of *E. coli* G92 during incubation with PS (ranging from 0 to 10 mM, **A**), ciprofloxacin (80 μg/mL) or in combination with PS (10 mM, **B**) within 1 h, CCCP was used as an efflux pump inhibitor.

**
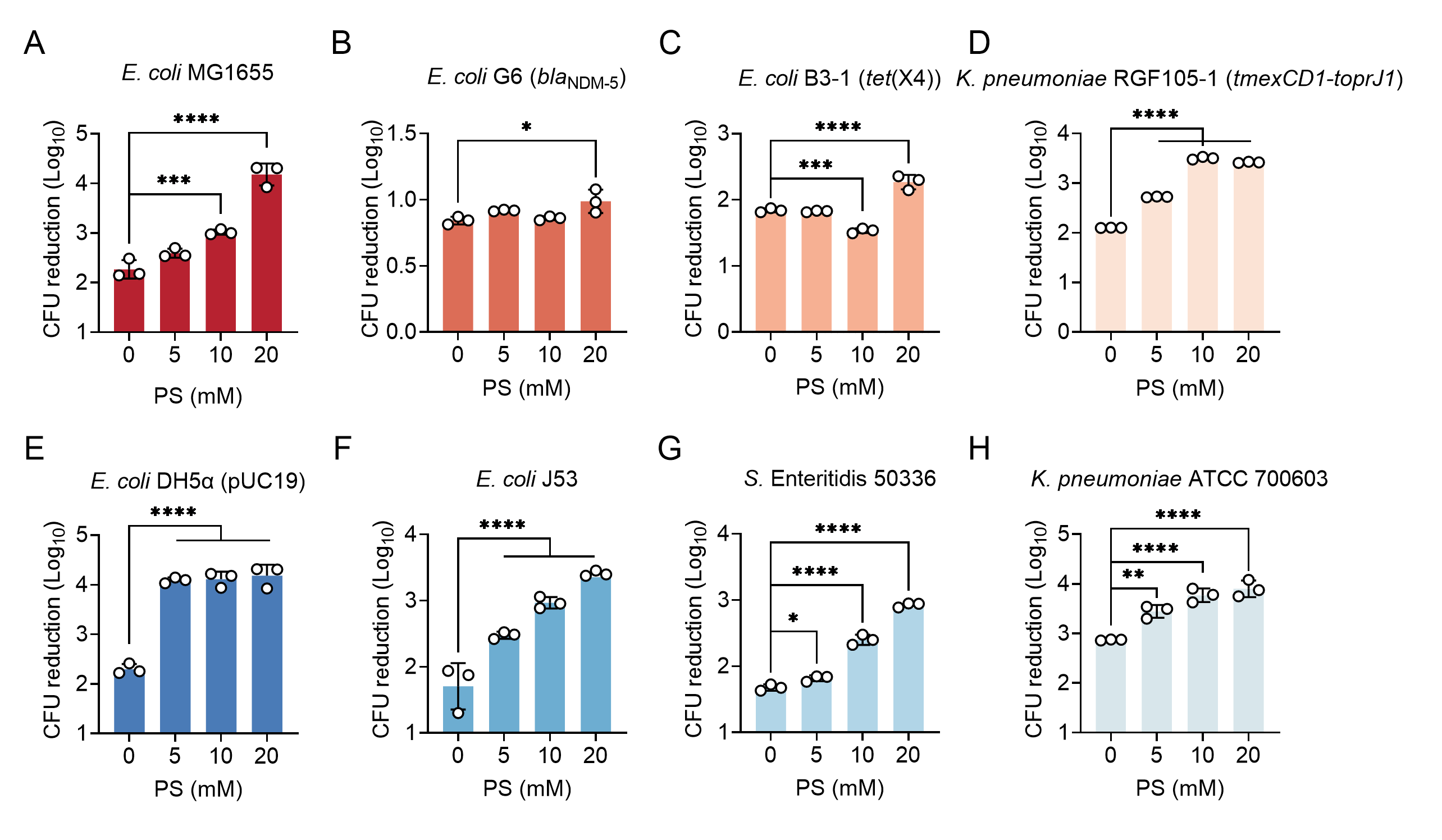
**

**Figure S10. Co-cultivation of *mcr-*negative bacteria with PS does not weakens its susceptibility to ciprofloxacin.**

CFU reduction (Log_10_) of *mcr-*negative bacteria cultured with PS (ranging from 0 to 20 mM) after treated with ciprofloxacin. All data from triplicate biological experiments were presented as means ± SD, and the significance determined by non-parametric one-way ANOVA. **P* < 0.05, ***P* < 0.01, ****P* < 0.001, *****P* < 0.0001.


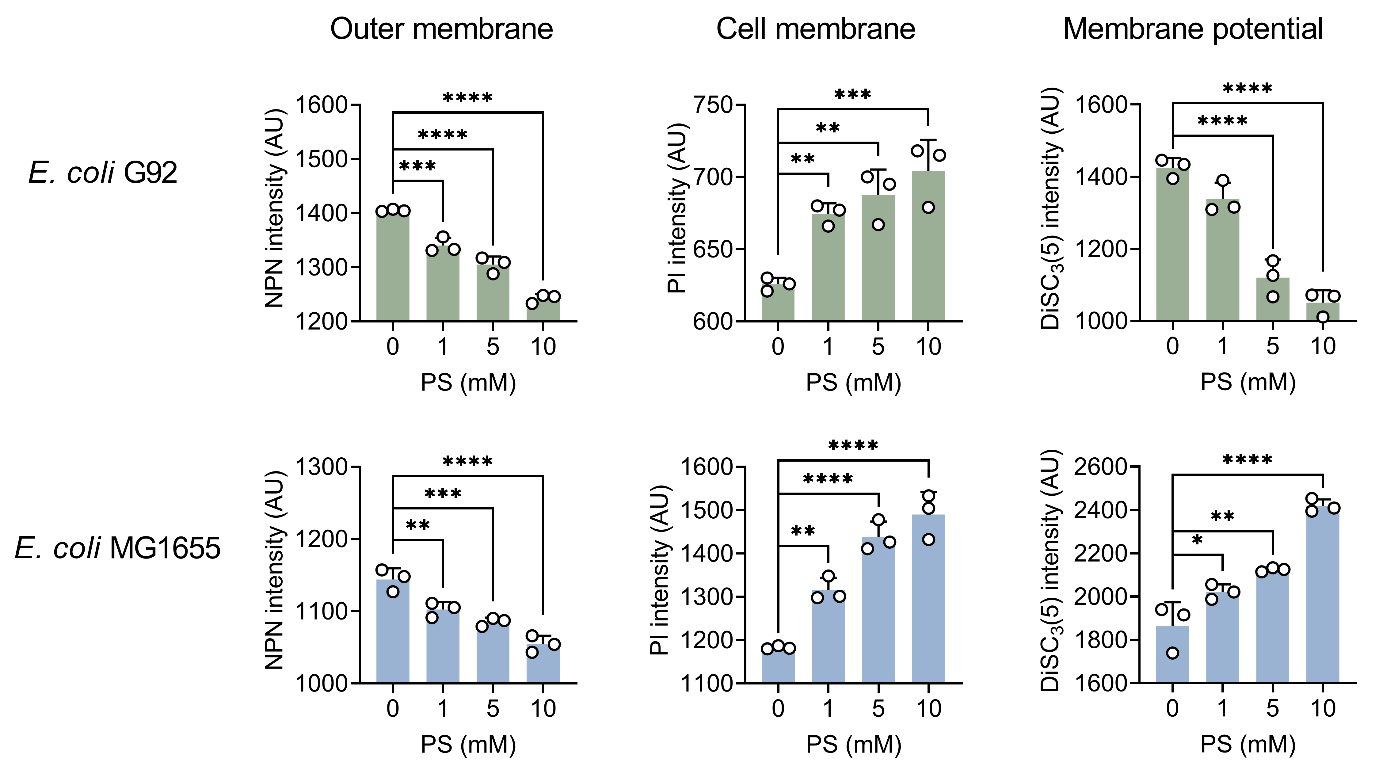


**Figure S11. Effects of PS on membrane permeability and membrane potential of *E. coli* G92 and *E. coli* MG1655.**

The variation tendencies of *mcr-1*-positive and negative bacteria in outer membrane and cell membrane permeability upon PS treatment were consistent, while the trend in membrane potential was opposite. All data from triplicate biological experiments were presented as means ± SD, and the significance determined by non-parametric one-way ANOVA. **P* < 0.05, ***P* < 0.01, ****P* < 0.001, *****P* < 0.0001.


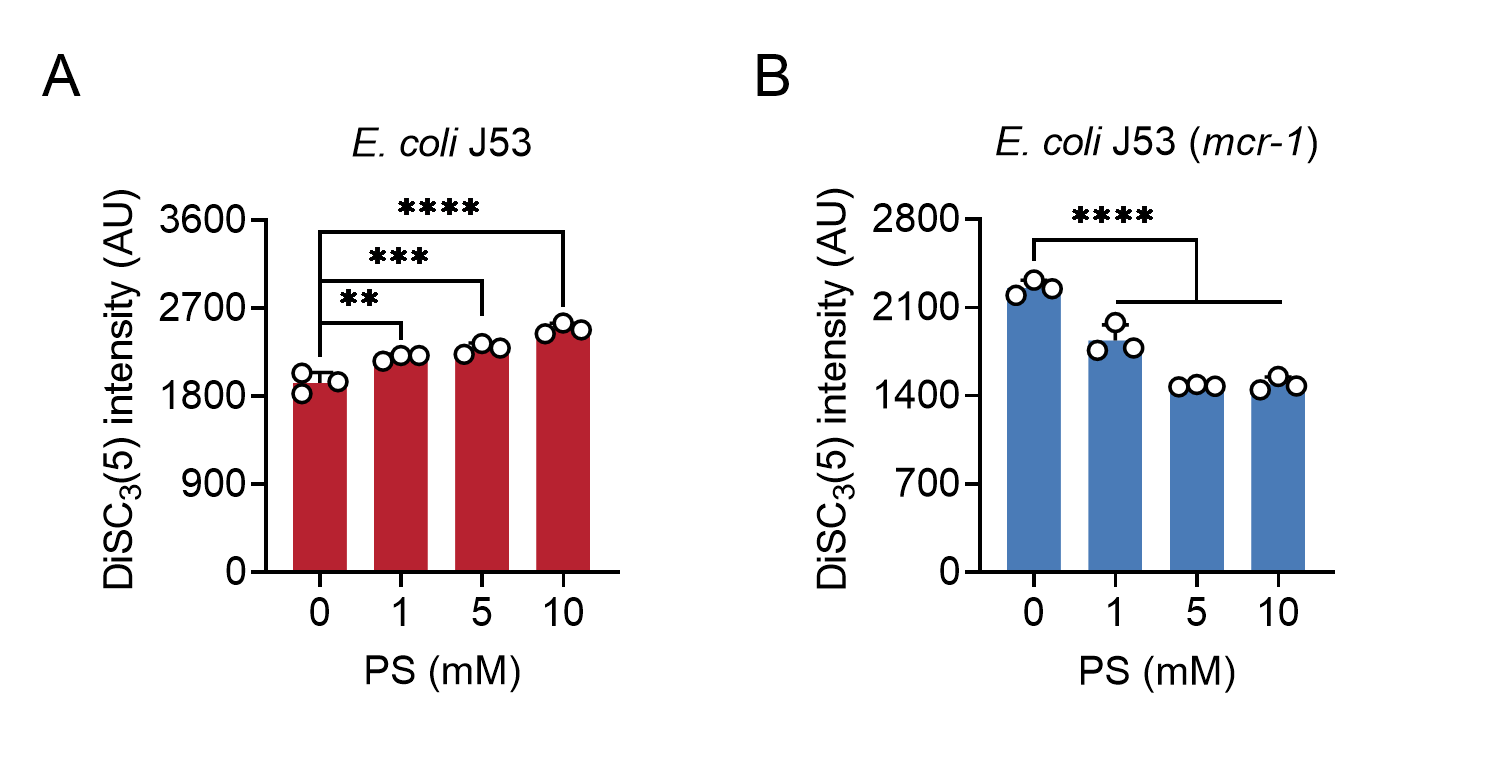


**Figure S12. Effects of PS on membrane potential of *E. coli* J53 and *E. coli* J53 (*mcr-1*).**

Membrane potential changes of *E. coli* J53 **(A)** and *E. coli* J53 (*mcr-1*) **(B)** treated by PS (ranging from 0 to 10 mM) was determined using fluorescence dye DiSC_3_(5). All data from triplicate biological experiments were presented as means ± SD, and the significance determined by non-parametric one-way ANOVA. ***P* < 0.01, ****P* < 0.001, *****P* < 0.0001.

**Tables**

**Table S1. Bacterial strains used in this study.**

| Strains | Source/References |
| --- | --- |
| *E. coli* MG1655 | In this study |
| *E. coli* B2 (*mcr-1* + *bla*_NDM-5_) | In this study |
| *E. coli* G92 (*mcr-1*) | In this study |
| *S. enterica* SC2016090 (*mcr-3*) | (1) |
| *K. pneumoniae* D120 (*mcr-8*) | (2) |
| *S.* Typhimurium sdj02 (*mcr-9*) | In this study |
| *E. hormaechei* K528 (*mcr-10*) | (3) |
| *E. coli* G6 (*bla*_NDM-5_) | In this study |
| *E. coli* B3-1 (*tet*(X4)) | In this study |
| *K. pneumoniae* RGF105-1 (*tmexCD1-toprJ1*) | In this study |
| *E. coli* J53 (*mcr-1*) | In this study |
| Δ*FRD* (*mcr-1*) | In this study |
| Δ*PTA-ackA* (*mcr-1*) | In this study |
| *E. coli* J53 | In this study |
| Δ*PTA-ackA*Δ*FRD* (*mcr-1*) | In this study |
| Δ*OmpF* (*mcr-1*) | In this study |
| *E. coli* DH5α (pUC19) | In this study |
| *E. coli* DH5α (pUC19-*mcr-1*) | In this study |
| *K. pneumoniae* ATCC 7000603 | ATCC |
| *S.* Enteritidis 50336 | (4) |

ATCC, American Type Culture Collection; MEM, meropenem; COL, colistin; TIG, tigecycline.

**References**

(1) R. Li *et al.*, The genomic epidemiology of *mcr*-positive *Salmonella enterica* in clinical patients from 2014 to 2017 in Sichuan, China and global epidemiological features. *J. Infect.*, **85**, 702-769 (2022).

(2) X. Yang *et al.*, Emergence of *mcr-8.2*-bearing *Klebsiella quasipneumoniae* of animal origin. *J. Antimicrob. Chemother.*, **74**, 2814-2817 (2019).

(3) Y. Yin *et al.*, Emergence and transmission of plasmid-mediated mobile colistin resistance gene in humans and companion animals. *Microbiol. Spectr.*, **10**, e0209722 (2022).

*(4)* Meng X *et al.,* RyhB paralogs downregulate the expressions of multiple survival-associated genes and attenuate the survival of *Salmonella* Enteritidis in the chicken macrophage HD11. *Microorganisms* **11**, 214 (2023).

**Table S2. Minimum inhibitory concentration (MIC) analysis of PS against different pathogens used in this study.**

| Pathogens | MIC (mM) |
| --- | --- |
| *E. coli* MG1655 | >125 |
| *E. coli* B2 (*mcr-1* + *bla*_NDM-5_) | >125 |
| *E. coli* G92 (*mcr-1*) | >125 |
| *S. enterica* SC2016090 (*mcr-3*) | >125 |
| *K. pneumoniae* D120 (*mcr-8*) | >125 |
| *S.* Typhimurium sdj02 (*mcr-9*) | >125 |
| *E. hormaechei* K528 (*mcr-10*) | >125 |
| *E. coli* G6 (*bla*_NDM-5_) | >125 |
| *E. coli* B3-1 (*tet*(X4)) | >125 |
| *K. pneumoniae* RGF105-1 (*tmexCD1-toprJ1*) | >125 |
| *E. coli* J53 (*mcr-1*) | >125 |
| Δ*FRD* (*mcr-1*) | >125 |
| Δ*PTA-ackA* (*mcr-1*) | >125 |
| *E. coli* J53 | 125 |
| Δ*PTA-ackA*Δ*FRD* (*mcr-1*) | 125 |
| Δ*OmpF* (*mcr-1*) | 125 |
| *E. coli* DH5α (pUC19) | >125 |
| *E. coli* DH5α (pUC19-*mcr-1*) | >125 |
| *K. pneumoniae* ATCC 7000603 | >125 |
| *S.* Enteritidis 50336 | >125 |

**Table S3. Antibiotic susceptibility of the strain in this study.**

| Pathogens | Antibiotics | MIC (μg/mL) |
| --- | --- | --- |
| *E. coli* MG1655 | Ciprofloxacin | <0.25 |
| *E. coli* B2 (*mcr-1* + *bla*_NDM-5_) | Ciprofloxacin | 8 |
| *E. coli* G92 (*mcr-1*) | Ciprofloxacin | 8 |
| *S. enterica* SC2016090 (*mcr-3*) | Ciprofloxacin | <0.25 |
| *K. pneumoniae* D120 (*mcr-8*) | Ciprofloxacin | <0.25 |
| *S.* Typhimurium sdj02 (*mcr-9*) | Ciprofloxacin | <0.25 |
| *E. hormaechei* K528 (*mcr-10*) | Ciprofloxacin | <0.25 |
| *E. coli* G6 (*bla*_NDM-5_) | Ciprofloxacin | 8 |
| *E. coli* B3-1 (*tet*(X4)) | Ciprofloxacin | 16 |
| *K. pneumoniae* RGF105-1 (*tmexCD1-toprJ1*) | Ciprofloxacin | 16 |
| *E. coli* B2 (*mcr-1* + *bla*_NDM-5_) | Levofloxacin | 16 |
| *E. coli* B2 (*mcr-1* + *bla*_NDM-5_) | Norfloxacin | 16 |
| *E. coli* B2 (*mcr-1* + *bla*_NDM-5_) | Ampicillin | >256 |
| *E. coli* B2 (*mcr-1* + *bla*_NDM-5_) | Colistin | 2 |
| *E. coli* B2 (*mcr-1* + *bla*_NDM-5_) | Gentamicin | 32 |
| *E. coli* B2 (*mcr-1* + *bla*_NDM-5_) | Tetracycline | 64 |
| *E. coli* J53 (*mcr-1*) | Ciprofloxacin | <0.25 |
| Δ*FRD* (*mcr-1*) | Ciprofloxacin | <0.25 |
| Δ*PTA-ackA* (*mcr-1*) | Ciprofloxacin | <0.25 |
| *E. coli* J53 | Ciprofloxacin | <0.25 |
| Δ*PTA-ackA*Δ*FRD* (*mcr-1*) | Ciprofloxacin | <0.25 |
| Δ*OmpF* (*mcr-1*) | Ciprofloxacin | <0.25 |
| *E. coli* DH5α (pUC19) | Ciprofloxacin | <0.25 |
| *E. coli* DH5α (pUC19-*mcr-1*) | Ciprofloxacin | <0.25 |
| *K. pneumoniae* ATCC 7000603 | Ciprofloxacin | 0.5 |
| *S.* Enteritidis 50336 | Ciprofloxacin | <0.25 |

**Table S4. PS induction has no effect on the MIC values.**

| Pathogens | MIC (μg/mL)^a^ | MIC (μg/mL)^b^ |
| --- | --- | --- |
| *E. coli* B2 (*mcr-1* + *bla*_NDM-5_) | 16 | 16 |
| *E. coli* G92 (*mcr-1*) | 8 | 8 |
| *S. enterica* SC2016090 (*mcr-3*) | <0.25 | <0.25 |
| *K. pneumoniae* D120 (*mcr-8*) | <0.25 | <0.25 |
| *S.* Typhimurium sdj02 (*mcr-9*) | <0.25 | <0.25 |
| *E. hormaechei* K528 (*mcr-10*) | <0.25 | <0.25 |

**^a^** Initial MIC of pathogens against ciprofloxacin

**^b^** MIC analysis of ciprofloxacin against tested pathogens after co-cultured with PS for 4 h

**Table S5. PS induction does not affect MIC values of tolerance strains.**

| Antibiotics | MIC (μg/mL)^a^ | MIC (μg/mL)^b^ |
| --- | --- | --- |
| Colistin | 2 | 4 |
| Gentamicin | 32 | 32 |
| Meropenem | 16 | 16 |
| Ciprofloxacin | 16 | 16 |
| Levofloxacin | 8 | 16 |

**^a^** Initial MIC of *E. coli* B2 against antibiotics

**^b^** Isolated tolerance strains from TDtest against antibiotics

**Table S6. Primers for RT-qPCR analysis in this study.**

| Genes | Primers | Sequence (5'-3') |
| --- | --- | --- |
| *fumB* | Forward | CGCTTCTTTTATGCTCCGCC |
|  | Reverse | GTCGTTTTCGCTGGCTTCTG |
| *frdA* | Forward | TCAGAACTGTGAAACCCGCA |
|  | Reverse | CAGTTCCGCCAGGGAGTTAG |
| *frdD* | Forward | GGTCGCGTATTCCTGTTCCT |
|  | Reverse | GGATAGCAGCCAGACCGTAG |
| *dcuB* | Forward | TGATCCGGCATACATCGTGG |
|  | Reverse | TGTGGTTGATGACGAAGCGA |
| *nuoA* | Forward | CCAAAACCTTCGGCAGTGTG |
|  | Reverse | TGAGACGATTTCGCCAGCAT |
| *cyoB* | Forward | ATCCTGCCTGTTTTCGGTGT |
|  | Reverse | GTGATACAGACGGTTGCCCA |
| *cyoC* | Forward | GCCCGACAGGTAAGGACATT |
|  | Reverse | AACGCCAGCCAGGAGATAAC |
| *cyoD* | Forward | CGGCTGTTTCTGCCAACTTC |
|  | Reverse | GTTCCATGTTGACGCTTCGG |
| *cpxR* | Forward | TAAGCCAGGAAGTGTTGGGC |
|  | Reverse | GCAGTTTACGACGCAGGTTG |
| *ompF* | Forward | GGCTTTGGTATCGTTGGTGC |
|  | Reverse | TTCGCGTCGTACTTCAGACC |
| *pta* | Forward | TCGCTAAAACGCTGAATGCG |
|  | Reverse | ATAACGCCGGTGATGTTGGT |
| *ackA* | Forward | GGAAGCGGCAAAAATGCTGA |
|  | Reverse | TGCGGATAGCAGAAACGGAA |
| *sdhA* | Forward | CGGCTGTTTCTGCCAACTTC |
|  | Reverse | GTTCCATGTTGACGCTTCGG |
| *sdhB* | Forward | GATTTCGGCACTCAACCAGC |
|  | Reverse | TGTCTACCACCAAATCGCGG |
| *sucA* | Forward | GCAGTTTGCTCACGTCAAGG |
|  | Reverse | TCACGGAAATGATGCTGGCT |
| *sucB* | Forward | GGTGTGTTCGGTTCCCTGAT |
|  | Reverse | CGTGCATACCCAGAATTGCG |
| *sucD* | Forward | CATCGACGCAGGCATCAAAC |
|  | Reverse | GCTTCACTTTCACGGTCAGC |
| *lexA* | Forward | AATTGTTTCCGGCGCATCAC |
|  | Reverse | AATACGTGCGACAACGACCT |
| *recB* | Forward | CCTGCGTGGCGTTGATAAAG |
|  | Reverse | TCCATCAGGGCAATCAACCC |
| *recC* | Forward | CTGCTGGCTTCATGGGGTAA |
|  | Reverse | CGTCATCTATGCCCCAACGA |
| *recD* | Forward | TTGATTTTGCCGAGCCAACG |
|  | Reverse | GAACTAAACAACGCCGCCAG |
| *dinI* | Forward | GCGAAAACTTCTCCATTGCCA |
|  | Reverse | GCATACTGAATACGGCGGGA |
| *ssb* | Forward | GGCAATATCGGTGGTGGTCA |
|  | Reverse | GCTGAACTGATTGCCACCCT |
| *sulA* | Forward | AGGCATCTGGGCTACCCTTA |
|  | Reverse | CGTAAAGCGCGAACCATTGA |
| *dps* | Forward | CCAAACAAGCGCACTGGAAC |
|  | Reverse | GGGTAACTTTTCAGCGGGGT |
| *sodA* | Forward | GAAAGGCGATAAACTGGCGG |
|  | Reverse | GCGCCAGAAATAGCTTCACC |
| *sodB* | Forward | ATCACTACGGCAAGCACCAT |
|  | Reverse | GCCAAAAGATGCGGCGATAG |
| *16S rRNA* | Forward | CCTACGGGAGGCAGCAG |
|  | Reverse | ATTACCGCGGCTGCTGG |

**Table S7. Isolated tolerance strains from TDtest analyzed by whole genome sequencing.**

| Position/Nucleotide change | Protein change | Annotation |
| --- | --- | --- |
| 614 / G>C | Trp205Ser | VgrG protein |
| 143 / A>C | Gln48Pro | Phage protein |
| 395 / T>A | Leu132Gln | Phage protein |
| 413 / T>C | Leu138Ser | Phage protein |
| 430 / T>G | Leu144Val | Phage protein |
| 473 / C>T | Ser158Phe | Phage protein |
| 872 / C>T | Pro291Leu | Translation elongation factor Tu |
| 461 / T>C | Ile154Thr | Translation elongation factor Tu |
| 155 / T>G | Leu52Arg | Translation elongation factor Tu |
| 832 / A>T | Ile278Phe | hypothetical protein |
| 261 / A>G | Ter87Trpext*? | Phage tail fiber assembly protein YcfA |
| 557 / G>C | Arg186Pro | Phage tail tip, host specificity protein J |
| 122 / C>T | Ala41Val | IS1 protein InsA |
| 206 / T>C | Leu69Pro | hypothetical protein |
| 132 / A>C | Leu44Phe | Transposase InsI for insertion sequence element IS30 |
| 983 / C>T | Thr328Ile | Dienelactone hydrolase family protein |
| 392 / T>C | Leu131Ser | H repeat-associated protein, YhhI family |
| 134 / T>G | Ile45Arg | core protein |
| 145 / A>G | Thr49Ala | Phage tail fiber, tail fiber assembly protein Tfa |
| 218 / A>T | Glu73Val | Phage tail fiber, tail fiber assembly protein Tfa |
| 209 / T>C | Ile70Thr | Phage tail fiber, tail fiber assembly protein Tfa |
| 161 / C>T | Ser54Leu | Phage tail fiber, tail fiber assembly protein Tfa |
| 152-155 / delGGCA insTGCT | ArgGln50-51LeuLeu | Phage tail fiber, tail fiber assembly protein Tfa |
| 140 / G>A | Arg47His | Transposase InsH for insertion sequence element IS5 |
| 715 / G>C | Gly239Arg | Transposase InsH for insertion sequence element IS5 |
| 740 / A>G | Tyr247Cys | core protein |
| 248 / G>A | Cys83Tyr | core protein |
| 254 / A>G | Asp85Gly | Transposase InsF for insertion sequence IS3 |
